# A Bayesian deep segmentation framework for glioblastoma tumor segmentation using follow-up MRIs

**DOI:** 10.3389/fnimg.2025.1630245

**Published:** 2025-10-23

**Authors:** Tanjida Kabir, Kang-Lin Hsieh, Luis Nunez, Yu-Chun Hsu, Juan C. Rodriguez Quintero, Octavio Arevalo, Kangyi Zhao, Jay-Jiguang Zhu, Roy F. Riascos, Mahboubeh Madadi, Xiaoqian Jiang, Shayan Shams

**Affiliations:** ^1^Department of Health Data Science and Artificial Intelligence, McWilliams School of Biomedical Informatics, University of Texas Health Science Center at Houston, Houston, TX, United States; ^2^Center for Secure Artificial Intelligence For HealthCare, McWilliams School of Biomedical Informatics, University of Texas Health Science Center at Houston, Houston, TX, United States; ^3^Department of Diagnostic and Interventional Imaging, University of Texas Health Science Center at Houston, Houston, TX, United States; ^4^Department of Neurosurgery, University of Texas Health Science Center at Houston, Houston, TX, United States; ^5^Department of Radiology, Louisiana State University Health Sciences Center, Shreveport, LA, United States; ^6^Department of Statistics, University of Pittsburgh, Pittsburgh, PA, United States; ^7^Department of Clinical and Health Informatics, McWilliams School of Biomedical Informatics, University of Texas Health Science Center at Houston, Houston, TX, United States

**Keywords:** glioblastoma, magnetic resonance imaging, Bayesian deep learning, machine learning, brain tumor segmentation

## Abstract

**Background:**

Glioblastoma (GBM) is the most common malignant brain tumor with an abysmal prognosis. Since complete tumor cell removal is impossible due to the infiltrative nature of GBM, accurate measurement is paramount for GBM assessment. Preoperative magnetic resonance images (MRIs) are crucial for initial diagnosis and surgical planning, while follow-up MRIs are vital for evaluating treatment response. The structural changes in the brain caused by surgical and therapeutic measures create significant differences between preoperative and follow-up MRIs. In clinical research, advanced deep learning models trained on preoperative MRIs are often applied to assess follow-up scans, but their effectiveness in this context remains underexplored. Our study evaluates the performance of these models on follow-up MRIs, revealing suboptimal results. To overcome this limitation, we developed a Bayesian deep segmentation model specifically designed for follow-up MRIs. This model is capable of accurately segmenting various GBM tumor sub-regions, including FLAIR hyperintensity regions, enhancing tumor areas, and non-enhancing central necrosis regions. By integrating uncertainty information, our model can identify and correct misclassifications, significantly improving segmentation accuracy. Therefore, the goal of this study is to provide an effective deep segmentation model for accurately segmenting GBM tumor sub-regions in follow-up MRIs, ultimately enhancing clinical decision-making and treatment evaluation.

**Methods:**

A novel deep segmentation model was developed utilizing 311 follow-up MRIs to segment tumor subregions. This model integrates Bayesian learning to assess the uncertainty of its predictions and employs transfer learning techniques to effectively recognize and interpret textures and spatial details of regions that are typically underrepresented in follow-up MRI data.

**Results:**

The proposed model significantly outperformed existing models, achieving DSC scores of 0.833, 0.901, and 0.931 for fluid attenuation inversion recovery hyperintensity, enhancing tumoral and non-enhancing central necrosis, respectively.

**Conclusion:**

Our proposed model incorporates brain structural changes following surgical and therapeutic interventions and leverages uncertainty metrics to refine estimates of tumor, demonstrating the potential for improved patient management.

GitHub link: https://github.com/tanjidakabir/GBM_code

## 1 Introduction

Glioblastoma (GBM) is the most common, aggressive, and lethal primary malignant brain tumor in adults, with 12,000 new cases diagnosed annually in the United States (Senders et al., 2020). The median overall survival is 14.6–20.9 months for patients enrolled in clinical trials and 11 months for the real-world GBM population (Hohmann et al., 2017; Zhu et al., 2017). Magnetic resonance imaging (MRI) is the most common imaging modality for brain tumor patients in both standard of care (SOC) and clinical trials due to its wide availability and distinct visualization of the brain’s anatomical structures (Bernstock et al., 2022). Patients diagnosed with GBM receive multiple MRIs: one or more before craniotomy, one within the first 72 h post-operation, and multiple follow-up MRIs. The follow-up MRI is about 4 weeks after concomitant treatment with external beam radiation therapy (XRT) and temozolomide (TMZ). Subsequent MRIs are performed every two to 3 months for GBM status assessment (Stupp et al., 2017; Tan et al., 2020).

Preoperative MRIs are essential for initial diagnosis, identifying the tumor’s location and extent of the disease, which aids in surgical planning. Postoperative MRIs provide immediate feedback on the success of the surgical intervention, but they contain surgically induced contrast enhancements, which can lead to difficulties distinguishing between post-surgical changes (such as swelling, hemorrhage, or damage to healthy tissue) and residual tumor tissue (Rykkje et al., 2023). Therefore, brain tumor-treating physicians use follow-up MRIs to measure residual disease, determine tumor responses to treatment, detect tumor recurrence, and identify treatment-associated side effects in SOC and clinical trials (Ellingson et al., 2017). An accurate tumor assessment in follow-up MRI examinations is crucial for providing optimal care to GBM patients and for determining the efficacy of tested drugs or devices in clinical trials (Delgado-López and Corrales-García, 2016).

Manual estimation of tumor sizes is difficult, time-consuming, operator-dependent, and error-prone due to the irregularity of tumor contours and the potential for tumor infiltration into complex brain structures. Additionally, increased T1 signal changes in the surgical bed and surrounding areas can be misleading, particularly in postoperative and post-radiation (XRT) follow-up MRIs (Shukla et al., 2018), due to the presence of blood products, surgical debris, or post-radiation changes. In addition, some tumor regions may exhibit an infiltrative growth pattern that is not initially enhancing on MRIs (Zinn et al., 2011; Rao et al., 2016). Moreover, the tumors’ irregular shape, heterogeneous structure, tumor progression, and pseudo-progression (including craniotomy-related ischemic changes and radiation necrosis) complicate GBM evaluation, even for experienced neuro-radiologists (Arevalo et al., 2019). This challenge is particularly critical in patients with high-grade gliomas, as residual areas of enhancement have been shown to correlate with survival (Molinaro et al., 2020). Furthermore, intra- and inter-rater variability in glioma tumor boundary estimation has been reported as 20 and 28%, respectively (Mazzara et al., 2004). This variability underscores the need for automated segmentation models in clinical settings, an ongoing unmet need in the neuro-oncology community.

Accurate segmentation of GBM is essential for effective treatment planning, monitoring, and prognosis. Precise delineation of tumor boundaries enables targeted surgical resection, maximizing tumor removal while preserving healthy tissue—an essential factor in maintaining neurological function. Segmentation also plays a critical role in assessing treatment response and detecting recurrence on follow-up MRIs, allowing clinicians to identify subtle changes in tumor size or characteristics over time (BRATS, 2015). Additionally, it provides valuable insights into tumor shape, size, and subregions, which are important predictors of patient survival and increasingly inform personalized treatment strategies (Kickingereder et al., 2016).

In the past few decades, several deep learning models have demonstrated exemplary performance in the medical domain, leading to a growing research trend in brain tumor segmentation. However, most of these models have focused primarily on preoperative MRIs, and their performance has not been evaluated on follow-up MRIs (Akkus et al., 2015; Ghaffari et al., 2020). Helland et al. developed a deep segmentation model for early postoperative MRIs, but their Dice similarity score was lower than that of the standard preoperative segmentation models (Helland et al., 2023). Only one software, BraTumIA, was trained and tested on a combination of preoperative, postoperative, and follow-up MRIs. However, its performance on postoperative and follow-up MRIs was inferior to its performance on preoperative MRIs (Meier et al., 2016). The only FDA-approved deep segmentation model, VBrain Longitudinal, was trained and tested on brain metastases using both MRI and computed tomography (CT), but its performance on follow-up MRIs has not been reported (Hu et al., 2019). Additionally, Khalaf et al. highlighted reproducibility issues with the BraTumIA software, showing that it failed to accurately measure the enhancement region from MRIs acquired just 2 days apart (Abu Khalaf et al., 2021). Despite the lack of scientific evaluation regarding the generalizability and performance of these models on follow-up MRIs, some automated preoperative MRI-based segmentation software is still used for tumor measurements and treatment effect assessments in clinical research settings (Zhu et al., 2012; Porz et al., 2014; Fyllingen et al., 2016).

Deep segmentation models typically produce point-based predictions without accounting for the associated uncertainty. This lack of uncertainty awareness presents a significant challenge, as it can lead to models making overly confident, yet potentially incorrect, predictions on unseen data. Such confidence overlooks uncertainties arising from noisy data collection or those introduced during the modeling phase (Gawlikowski et al., 2021). In the context of GBM assessment, the model is likely to encounter test examples that differ substantially from the training data, which can result in unreliable predictions in certain cases. Incorporating uncertainty into the model’s outputs can help neuro-radiologists make more informed decisions regarding the reliability of these predictions. Among the various methods for estimating uncertainty, Bayesian learning is a well-established and effective approach for quantifying uncertainty mathematically (Gal and Ghahramani, 2016).

In this study, we have developed a comprehensive segmentation model named GBSUN (GlioBlastoma Segmentation and Uncertainty EstimatioN), specifically designed to delineate various GBM tumor subregions using follow-up MRI scans based on their distinct imaging characteristics. The GBSUN model accurately identifies different tumor areas, such as Fluid Attenuation Inversion Recovery (FLAIR) Hyperintensity Regions (FHR), Enhancing Tumor Regions (ER), and Non-Enhancing Central Necrosis Regions (NENR), while accounting for changes in brain and tumor structure post-surgery. Our framework builds on the original 3D U-Net model, enhanced with transfer learning and uncertainty estimation to improve performance.

GBM patients typically experience tumor recurrence during SOC treatment, with a median time of 7 months from diagnosis (Roger Stupp et al., 2005). Accurately diagnosing GBM at recurrence is challenging. When abnormal enhancement occurs outside the radiation field, standard MRI reliably identifies it as GBM progression. However, standard MRI struggles to differentiate between true tumor progression and radiation-induced necrosis (pseudo progression) when new or expanding enhancement is observed within the radiation field. Additionally, tumor recurrence in follow-up MRIs is often presented as small lesions, with very few cases showing non-enhancing regions. In our dataset, there are 47 cases of non-enhancing regions among 311 total cases. Follow-up MRIs generally show fewer non-enhancing necrotic regions due to surgical removal and post-surgical healing. Neurosurgeons aim to remove as much of the tumor and necrotic tissue as possible during surgery, ensuring maximal excision of pathological tissue. Non-enhancing regions, often necrotic parts of the tumor, are typically included in the excision. As a result, less necrotic tissue remains in follow-up MRIs post-surgery. Furthermore, after tumor resection, the brain begins to heal, and any remaining necrotic tissue may shrink or become less visible over time, further contributing to the reduction of non-enhancing regions (Kessler and Bhatt, 2018). To address the challenge of segmenting NENR regions, which are less frequently represented in follow-up MRI data, we employed a transfer learning approach (Torrey and Shavlik, 2010). Transfer learning allows the model to leverage knowledge from pre-trained models on preoperative images, enhancing its ability to detect non-enhancing regions and improving NENR segmentation performance in follow-up MRIs.

Additionally, we employed Bayesian learning to refine and enhance the predictive confidence of the proposed model. This approach also provides uncertainty estimation for various segmented areas, particularly around the tumor boundaries, where the risk of misclassification is highest. By integrating uncertainty information with transfer learning strategies, our model is better equipped to navigate the complexities inherent in follow-up MRI scans.

Finally, we enhanced our method by introducing case-specific threshold values for uncertainty calculations to minimize false negatives. To establish these thresholds, we computed the mean and variance of the background pixels, which represent the predominant class in follow-up MRIs. By sampling from the posterior distribution of the model’s parameters through multiple runs, we calculated the mean and variance for the FHR, ER, NENR, and background classes for each pixel. We then compared the mean values to the threshold: if the mean value of any class exceeds the threshold, that class is assigned to the pixel.

To validate the superiority of the GBSUN model, we conducted two sets of comparative evaluations. The first analysis focused on benchmarking the segmentation accuracy of GBSUN against state-of-the-art (SOTA) models. The second comparison assessed how GBSUN’s uncertainty estimation approach compares to the Monte Carlo dropout (Papadopoulos and Yeung, 2001) technique, with the goal of enhancing the reliability, safety, and interpretability of the model’s predictions. We also demonstrated that SOTA models are insufficient for detecting GBM tumors in follow-up MRIs, highlighting the need for an improved model for follow-up evaluation.

Our contributions in this study can be summarized as follows:

- Development of a novel Bayesian 3D U-Net model to improve predictive confidence and capture uncertainty.
- Overcoming data limitations and enhancing model performance through transfer learning.
- Leveraging uncertainty information to identify and correct potential misclassification areas.
- Introducing case-specific threshold values for uncertainty calculations to minimize false negatives.
- Accounting for changes in brain and tumor morphology when detecting tumor subregions by capturing spatial relationships between tumor subregions and surrounding brain structures.
- Creation of the largest follow-up MRI dataset for GBM tumor detection.

## 2 Materials and methods

### 2.1 Dataset description

This study was approved by the institutional review board (HSC-MS-17-0047). Informed consent was waived, and data collection and storage followed local guidance. The current study focused on a prospectively maintained institutional database with more than 500 subjects with high-grade glial neoplasms. Patients with the following criteria were included in this study.

- A confirmed diagnosis of glioblastoma IDH-wildtype (Wild type—270, Mutant—19, Missing—22)
- Only adult subjects (≥18 years)
- All scans included four MRI sequences: T1-WI (T1), T1-WI + gadolinium (T1-Gd), T2-WI (T2), and T2-Fluid Attenuated Inversion Recovery (T2-FLAIR)
- Available pathology reports in the electronic medical record system
- Diagnosed between 2005 and 2022.

A total of 311 follow-up MRI scans were utilized in this study. These follow-up MRIs were acquired 4 weeks post-XRT and TMZ and every 2 months afterward. The initial, immediate postoperative MRIs were not used. We selected only one scan per subject, specifically the earliest scan after XRT-TMZ that met the inclusion criteria.

The dataset was randomly divided into 80, 10, and 10% for training, validation, and testing, respectively. Additionally, the cross-validation technique (e.g., 5-fold cross-validation) was utilized to ensure robust performance evaluation across multiple subsets of the data. This approach helps mitigate the risk of overfitting and ensures that the model’s performance is not dependent on a single train-test split. The 10% test set is randomly sampled to ensure it adequately reflects the diversity of the entire dataset. The age distribution across the training, validation, and test sets is consistent. The patients’ demographic information and age distribution are summarized in Supplementary Tables 1, 2.

### 2.2 Image acquisition, preprocessing, and annotation process

MRIs were acquired following an institutionally standardized brain tumor protocol using a 1.5 T or a 3.0 T scanner. The isovolumetric MPRAGE 3D T1-weighted images of the brain were acquired in the axial plane after intravenous administration of contrast. Multiplanar reformats with a slice thickness of 1 mm were obtained. Detailed information about the MRI acquisition parameters is provided below in Supplementary Tables 3A,B.

**Figure 1:**
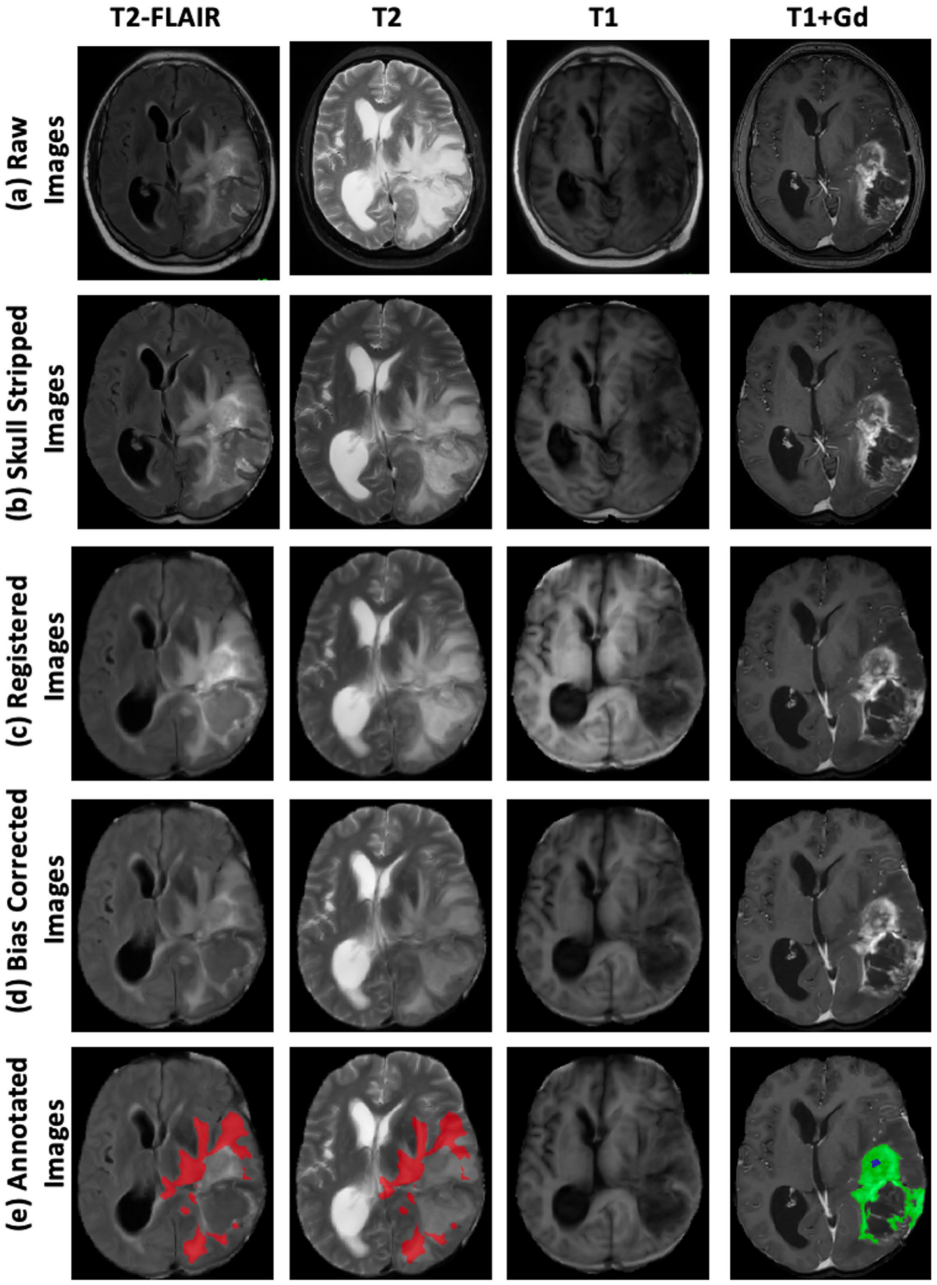
Step-by-step preprocessing pipeline for multi-modal brain MRI data. Columns display four MRI sequences: T2-FLAIR, T2-weighted, T1-weighted, and T1-weighted with gadolinium contrast enhancement (T1 + Gd). Rows illustrate sequential preprocessing steps: **(a)** Raw Images—original MRI scans acquired directly from the scanner; **(b)** Skull-Stripped Images—removal of non-brain tissues to isolate intracranial structures; **(c)** Registered Images—alignment of all modalities to a common spatial reference frame for voxel-wise correspondence; **(d)** Bias-Corrected Images—correction of intensity inhomogeneities to improve image uniformity and facilitate analysis; **(e)** Annotated Images—expert tumor labels overlaid on bias-corrected images, where Fluid Attenuation Inversion Recovery (FLAIR) Hyperintensity Regions (red), Enhancing Tumor Regions (green), and Non-Enhancing Central Necrosis Regions (blue).

The following steps—skull stripping, image registration, and bias correction—were performed to minimize the effects of varying magnetic fields and image resolution, as illustrated in Figures 1a–d.

- Skull stripping: The Simple Skull Stripping (S3) (Roy and Maji, 2015) method was used to remove the skull from all four MRI modalities. The S3 method uses the SRI24 template (Rohlfing et al., 2010) to estimate the brain area and create a mask to extract brain tissue.
- Image registration: FreeSurfer (Fischl, 2012) was employed to register the MRI scans using the SRI24 template, ensuring the data were geometrically aligned (Toga and Thompson, 2001). This step facilitates consistent anatomical alignment across the different imaging modalities. We employed FreeSurfer’s *MRICoreg* with a 12-degree-of-freedom affine transform to align each MRI scan to the SRI24 template. This configuration accounts for translations, rotations, scaling, and shear, thereby ensuring geometric consistency across subjects and scanners. Registration was performed with the following parameter settings: spatial scales of 2 and 4 voxels, a maximum of 4 iterations, function tolerance of 1.0e-07, line minimization tolerance of 1.0e-03, and a saturation threshold of 9.999e+01. The estimated transforms (.lta files) were subsequently applied using FreeSurfer’s *ApplyVolTransform*, which by default performs resampling with trilinear interpolation into the template space.
- Bias correction: N4 Bias Field Correction (Tustison et al., 2010) (SimpleITK) was applied to mitigate low-frequency intensity inhomogeneities introduced by scanner hardware and acquisition protocols. The N4 algorithm iteratively estimates a smooth multiplicative bias field and normalizes image intensities, thereby improving uniformity and enhancing the reliability of intensity-based feature learning. We used the default parameter settings of the *N4BiasFieldCorrection* function in SimpleITK: input pixel type = sitkFloat64, maximum number of iterations = 50 (per level), bias field full width at half maximum = 0.15, number of histogram bins = 200, mask label = 1, shrink factor = 4, and convergence threshold = 0.0.

Together, these steps harmonized data acquired on different MRI platforms and replicated the preprocessing philosophy of BraTS, ensuring comparability with benchmark datasets and reproducibility of our results.

In our study, we utilized the BraTS 2023 Adult Glioma dataset, which comprises clinically acquired, multi-institutional mpMRI scans across four sequences (T1, T1 + Gd, T2, and T2-FLAIR). For public release, all images are distributed as preprocessed NIfTI volumes, which have been co-registered to the SRI24 template, resampled to isotropic 1 mm^3^ resolution, and skull-stripped. As part of this preprocessing and de-identification pipeline, the original DICOM metadata are not available; therefore, scanner-specific acquisition parameters (e.g., field strength, TR, TE, flip angle) cannot be reported.

After image preprocessing, the MRIs were transferred to the neuro-radiology workstation for semi-automatic volumetric analysis and tumor segmentation. This analysis was conducted by a neuro-radiology researcher and a clinical fellow, with each case meticulously supervised by a board-certified neuroradiologist. ITK-SNAP (2019, version 3.8) was used to generate segmentation ground truth. Segmentation was carried out using an automatic region of interest (ROI) tool, which selects pixels within a specified signal intensity range. Once the automatic ROI was generated, the neuro-radiologists manually refined the ROIs, excluding areas incorrectly included in the volumetric analysis. Segmentation was performed across four MR sequences simultaneously. T1 and T1 + gadolinium (Gd) were used to segment the NENR and ER. T2 and T2-FLAIR were used to identify FHR. Each scan labeled three tumoral regions: FHR, ER, and NENR. Figure 1e illustrates a sample image with labeled regions.

We were not able to differentiate between pre- and post-treatment enhancement due to the diverse etiologies within each classification. Pre-treatment enhancement could be attributed to tumor, infection, or inflammation, whereas post-treatment enhancement could result from tumor progression, perioperative ischemic changes, and radiation necrosis.

### 2.3 Proposed follow-up model description

#### 2.3.1 Model overview

**Figure 2:**
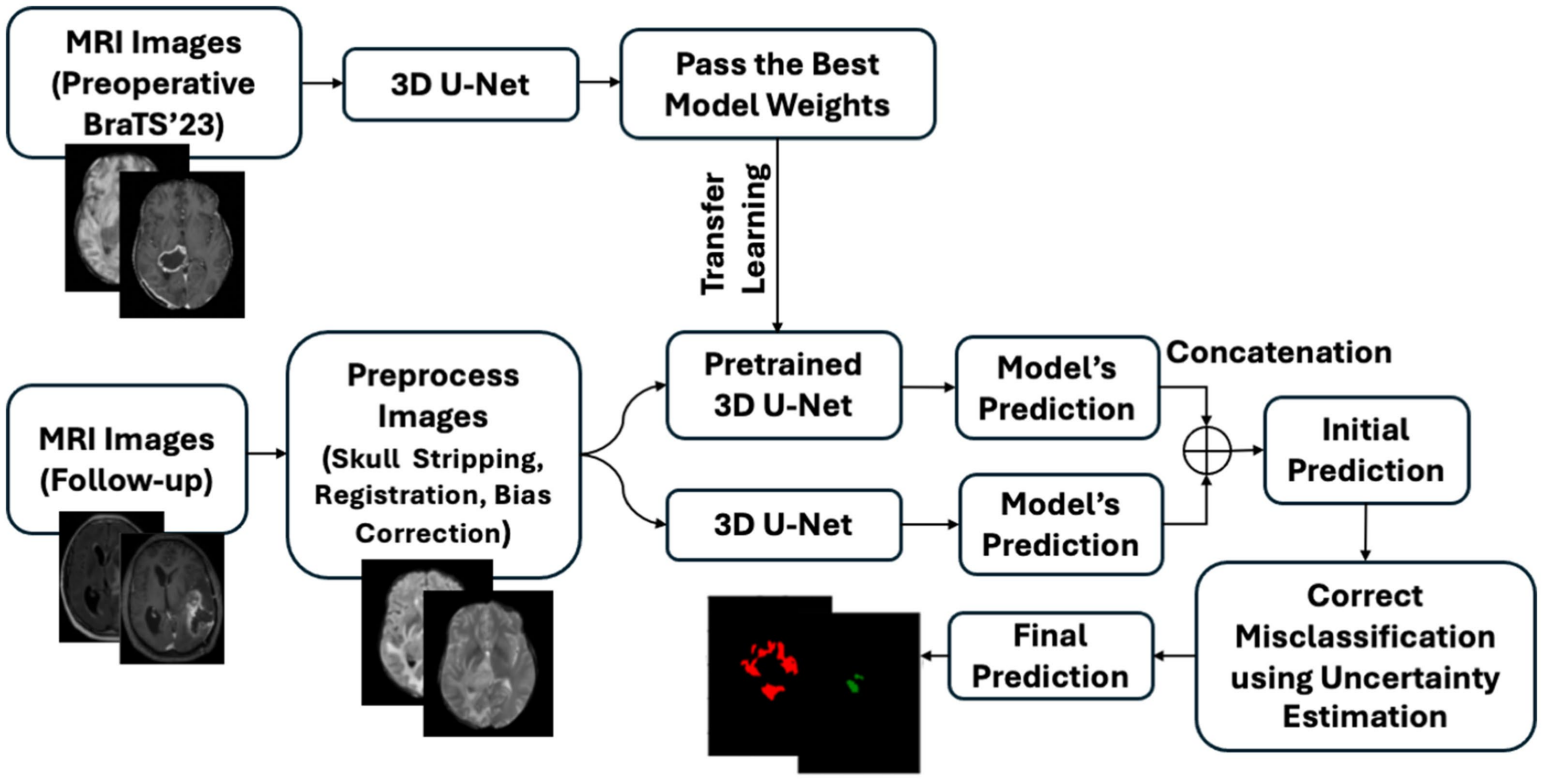
Overview of the proposed brain tumor segmentation framework. Preoperative BraTS’23 MRI scans are used to train a 3D U-Net, whose best weights initialize a pretrained model for follow-up MRI data. Follow-up scans undergo preprocessing steps before being segmented by both the pretrained and a newly trained 3D U-Net. Predictions from both models are combined and refined using uncertainty-based misclassification correction to generate the final segmentation.

Figure 2 illustrates the high-level architecture of the proposed framework, which aims to achieve high segmentation accuracy while maintaining interpretability. As shown, the segmentation model takes four preprocessed MRI sequences as input and generates an initial prediction. Transfer learning was utilized to further enhance model performance, particularly in the NENR region. Finally, misclassified pixels are corrected using uncertainty information.

#### 2.3.2 3D-Unet model architecture and input formats

**Figure 3:**
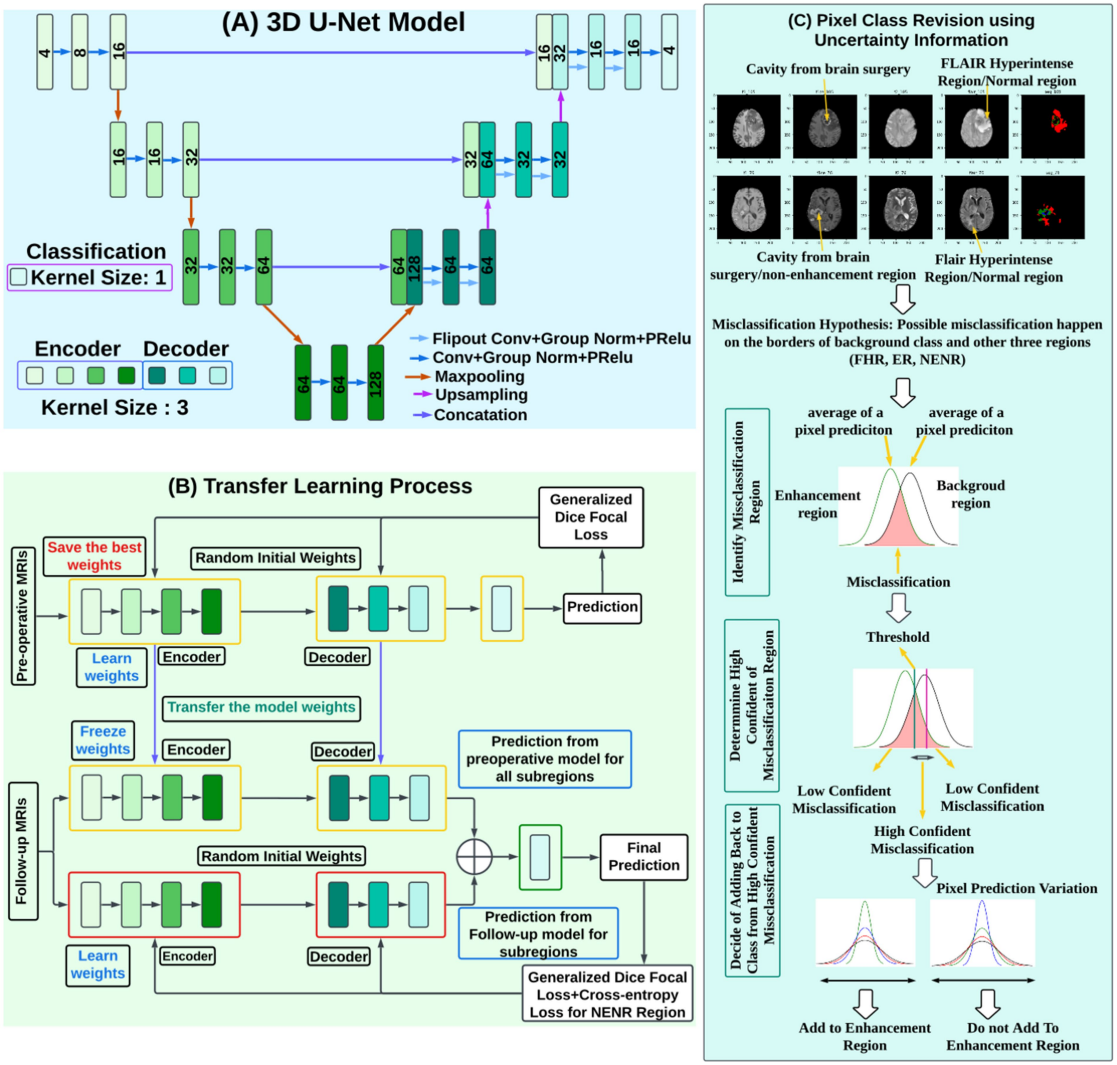
Detailed architecture and workflow of the proposed uncertainty-based brain tumor segmentation framework. **(A)** 3D U-Net model: The network follows an encoder–decoder structure with convolutional blocks, max-pooling for downsampling, and transposed convolutions for upsampling. Feature maps from the encoder are concatenated with the decoder via skip connections. **(B)** Transfer learning process: A 3D U-Net is first trained on preoperative MRI scans using generalized Dice focal loss, and the best weights are saved. For follow-up MRI scans, the encoder is initialized with pretrained weights and fine-tuned alongside a second 3D U-Net trained from scratch. Predictions from the pretrained and newly trained models are combined to produce the final segmentation. **(C)** Pixel class revision using uncertainty information: Potential misclassifications in the FHR, ER, and NENR are identified by analyzing pixel-wise uncertainty distributions. Misclassified pixels are categorized into high- and low-confidence errors, and pixel prediction variation guides whether a voxel is reassigned to the correct class.

The proposed GBSUN model was developed based on the 3D U-Net (Wang et al., 2019). Figure 3A illustrates the basic structure of the model, comprising three major components: the encoder, decoder, and classification layer. The encoder extracts features from the input data, and the decoder projects the embedded features extracted by the encoder onto the pixel space to produce the classification results. The classification layer assigns classes to each pixel. The encoder is composed of 3D convolution layers with a kernel size of 3, a stride of 1, and a dilation set to 1, along with max-pooling layers with a kernel size of 2. The decoder mirrors the encoder’s architecture, featuring upsampling and convolutional layers with the same parameter settings. (IntelLabs/bayesian-torch, 2022; Wen et al., 2018). The final classification layer has a kernel, stride, and dilation size of 1.

Both the pre-trained and follow-up models use an identical 3D U-Net encoder–decoder backbone, where the encoder channel progression is [4, 8, 16, 32]. Each encoder block expands the number of channels by a factor of four, resulting in a bottleneck embedding dimension of 128 channels in both models. Consequently, the output embeddings from the two models are already of the same dimensionality and can be fused directly without the need for additional projection layers. This design eliminates the risk of mismatched feature sizes during blending and ensures that the combined representation is well-defined.

Each MRI sequence is input into a separate CNN channel, as each sequence captures different tissue properties and provides unique, complementary information. This approach enhances feature representation, leading to more accurate diagnoses. Instead of relying on pre-extracted or manually defined features or patches, the model uses the entire MRI scan as input. This allows the model to process the full-resolution image, learning both spatial and contextual information necessary for accurate pixel-level classification. Furthermore, by processing the entire image, the model can capture the broader context and relationships between different regions, which is crucial for precise segmentation. This approach also preserves the spatial relationships between objects and features, which is essential for understanding how different areas of the image relate to one another.

#### 2.3.3 Loss function

Unlike standard deep neural networks that generate single-point estimates, Bayesian learning quantifies both epistemic and aleatoric uncertainty (Gal and Ghahramani, 2016; Kendall and Gal, 2017). Aleatoric uncertainty arises from inherent noise in the data, such as sensor artifacts or patient motion, and reflects variability in the observations that cannot be reduced even with more data. In contrast, epistemic uncertainty stems from limited knowledge of the model parameters; it is high when the training data are sparse or unrepresentative and decreases as more data are incorporated. Bayesian methods capture epistemic uncertainty by maintaining a posterior probability distribution over model parameters, rather than relying on a single fixed set, thereby enabling the model to express confidence that adapts with data availability. At the same time, aleatoric uncertainty is captured through the probabilistic likelihood function, which models the inherent randomness in the data by representing outputs as distributions rather than deterministic values. By jointly modeling these two types of uncertainty, Bayesian learning not only improves prediction reliability but also provides calibrated confidence estimates, helping to highlight regions of low reliability and enhancing interpretability for clinical tasks.

To build the Bayesian U-Net, all 3D convolutional layers in the decoder were replaced with Flipout 3D convolutional layers (IntelLabs/bayesian-torch, 2022; Wen et al., 2018). The Flipout 3D convolutional layer is an efficient method that decorrelates gradients by implicitly sampling pseudo-independent weight perturbations for each example’s latent space. The prior mean and variance for the Flipout layers were set to zero and one, respectively, while the posterior mean and variance were set to zero and three, respectively.

This method allows the model to simultaneously optimize two types of loss functions: the region-based loss (generalized dice focal loss) and the distribution loss (Kullback–Leibler (KL) divergence loss).


Total Loss=Generalized Dice Focal Loss+KLDivergence Loss

Generalized dice focal loss is a weighted sum of generalized dice loss (Sudre et al., 2017) (GDL) and focal loss (Zhu et al., 2019; Lin et al., 2020) (FL). For the three-class classification problem, the GDL can be defined as


$$GDL=1-2\frac{\overset{3}{\underset{l=1}{\sum }}w_{l}\underset{n}{\sum }r_{\ln}p_{\ln}}{\overset{3}{\underset{l=1}{\sum }}w_{l}\underset{n}{\sum }\left(r_{\ln}+p_{\ln}\right)}$$

Here, $r_{n}$ is the gold standard and $p_{n}$ is the predicted probabilistic map over N image elements. $w_{l}=\frac{1}{{\left(\overset{N}{\underset{n=1}{\sum }}r_{\ln}\right)}^{2}}$ , used to provide invariance to different label set properties, utilizes the correlation between dice score and region size. In the GDL, the contribution of each label is corrected by the inverse of its volume.

FL is a dynamically scaled cross-entropy loss that can down-weight the contribution of easy examples and put more focus on hard and misclassified examples automatically during model training. The FL is defined as


$$FL\;\left(p_{t}\right)=-{\left(1-p_{t}\right)}^{\gamma }\;\log\left(p_{t}\right)$$

Here γ>0 reduces the relative loss for well-classified examples ( $p_{t}>0.5$ ) and puts more weight on miss-classified examples. The γ is a learnable focusing parameter γ≥0 .

Overall, we can compute the generalized Dice focal loss as


$$Generalized\;Dice\;Focal\;Loss=\lambda_{GDL}*GDL+\lambda_{FL}*FL$$

$\lambda_{\mathrm{GDL}}$ is the weight of GDL, and $\lambda_{\mathrm{FL}}$ is the weight of FL.

KL divergence between the prior distribution, P and the posterior distribution, Q is defined as


$$KL\;Divergence\;\left(P\|Q\right)=\underset{x\in X}{\sum }P\left(x\right)\;\log\frac{P\left(x\right)}{Q\left(x\right)}$$

Minimizing the KL divergence between P and Q ensures that Q approximates P.

Thus, the proposed model can estimate epistemic and aleatoric uncertainties of each class (FHR, ER, NENR, and background) for each pixel. Transfer learning and uncertainty information were leveraged to improve model performance. The details of each process are provided below:

#### 2.3.4 Transfer learning

Transfer learning involves transferring knowledge from a related task to improve generalizability, especially when the available dataset is too small (Torrey and Shavlik, 2010). In our dataset, only 47 follow-up MRI cases contain NENR regions, which is insufficient for training a complex model such as 3D-UNet. Preoperative MRIs, however, typically have a higher incidence of NENR regions. Therefore, we trained a model using preoperative MRIs to learn the morphology and spatial characteristics of non-enhancing regions. The learned information was then transferred to the follow-up model for detecting NENR regions. Figure 3B illustrates the transfer learning process in the proposed model.

The preoperative model was trained by preoperative MRIs from the BraTS’23 datasets (BRATS, 2015; Bakas et al., 2017, 2018) and optimized by generalized dice focal loss (Figure 3B). The model with the lowest validation loss was saved for knowledge transfer. By freezing these weights during follow-up training, we ensured that the knowledge from the preoperative data was preserved, allowing the model to retain critical baseline features learned from pre-operative MRIs, especially for NENR regions.

In parallel, another model was trained with randomly initialized weights using institutional follow-up MRIs and optimized with both generalized dice focal loss and cross-entropy for the non-enhancing region (Figure 3B). Training this model from scratch on follow-up MRIs enables it to capture features specific to the follow-up MRIs, enhancing its ability to identify tumor regions and other changes unique to the post-surgical context. Finally, the embeddings from both the preoperative and follow-up models were combined in the final classification layer. By combining the outputs of both models in the final prediction layer, we leveraged the strengths of each: the preoperative model for baseline tumor characteristics and the follow-up model for post-treatment adaptations.

The epoch vs. loss graph and epoch vs. Dice similarity score curve for both training and validation data are provided in Supplementary Figures 1A,B, respectively.

#### 2.3.5 Utilizing uncertainty information

Follow-up MRIs contain surgical-related defects of the skull and brain parenchyma, including burr hole and tumor tissue removal, which results in a cavity and bone repositioning. These anatomical changes of the brain increase the complexity of measuring tumor subregions and signals in follow-up MRIs compared to preoperative MRIs. Additionally, tumor subregions in follow-up MRIs are often not contiguous, unlike in preoperative scans, and may be surrounded by either normal brain tissue or surgical cavities. Both normal brain tissue and surgical cavities are treated as background.

As a result, the background class becomes more dominant than the other three classes (FHR, ER, NENR), leading to potential overestimation. The error-prone region for predicting background distribution is larger than for the other tumor regions for a given pixel, increasing the likelihood of misclassification between these three classes and the background.

To address the issue of misclassification of pixels as background, we utilize pixel uncertainty to improve the model’s performance. The following steps are employed when a pixel is misclassified as the background class:

1 Model Outputs: From a single run of the model, each pixel receives four probability scores corresponding to four classes: $p_{f}$ for FHR, $p_{e}$ for ER, $p_{n}$ for NENR, and $p_{b}$ for the background class_._ From multiple runs of the model, we gather a distribution $d_{f}\left(\mu_{f},\sigma_{f}^{2}\right)$ , $d_{e}\left(\mu_{e},\sigma_{e}^{2}\right)$ , $d_{n}\left(\mu_{n},\sigma_{n}^{2}\right)$ , and $d_{B}\left(\mu_{B},\sigma_{B}^{2}\right)$ . The uncertainty for each class is represented by the variance of its score distribution.
2 Threshold Calculation: We compute a threshold value using the background class’s mean ( $\mu_{B})$ and variance ( $\sigma_{B}^{2})$ .

$$threshold=\mu_{B}-\lambda \times \sigma_{B}$$

Where λ is a hyperparameter that controls the weight assigned to the variance. In our analysis the optimal value λ=0.1 was determined by tuning on the validation set. We have added Supplementary Table 5 to display different lambda values and their corresponding dice similarity scores.

3 Misclassification Detection: If the mean ( $\mu_{f}$ , $\mu_{e}$ , $\mu_{n}$ ) of any other three classes exceeds the threshold, we consider that the pixel has been misclassified as background.
4 Pixel Reclassification: If only one class satisfies the condition in step 3, the pixel is reclassified from background to that class.
5 Resolving Ambiguity: If multiple classes satisfy the condition in step 3, the class with the lowest variance is chosen as the final classification.

Figure 3C illustrates the detailed process of utilizing uncertainty information to improve the model performance, where ER is misclassified as normal brain regions.

#### 2.3.6 Model training and hyperparameter details

To minimize domain confusion between pre- and post-operative MRI, we first trained on pre-operative scans, where non-enhancing necrotic regions (NENR) are more consistently represented, and then fine-tuned on post-operative scans to adapt to tissue changes after resection. This sequential strategy reduces overfitting to one domain and improves robustness in distinguishing true tumor tissue from post-surgical alterations. We employed the Adam optimizer with a learning rate of 0.001 and a weight decay of 1e-5. The batch size was set to 8. The details of hyperparameters are mentioned in Supplementary Table 4. All experiments were conducted on an NVIDIA Tesla A100-SXM4-40GB platform (CUDA 12.7; PyTorch 1.14.0a0 + 44dac51). Model training required approximately 70 h for 100 epochs with a batch size of 8. The inference time for a single patient is approximately 1 min for 1,000 stochastic forward path using a server with 16 NVIDIA Tesla A100-SXM4-40GB GPU (CUDA 12.7).

### 2.4 Performance evaluation matrices

The Dice Similarity Coefficient (DSC) measures the spatial overlap between the model’s prediction and the ground truth. It is used to evaluate the segmentation results in terms of accuracy and generalizability.


$$DSC=\frac{2\times the\;Area\;of\;Overlap}{Total\;number\;of\;pixels\;in\;both\;images}$$

The Jaccard Index (JI) is a metric used to compare the similarity and diversity between the predicted and ground truth segments. It is defined as the size of the intersection of the two sets divided by the size of their union.


$$JI=\frac{Area\;of\;Overlap}{Area\;of\;Union}$$

Hausdorff distance (HD) is the maximum distance from any point in one set to the nearest point in the other set. Specifically, for two sets of points, X and Y , the Hausdorff distance is defined as:


$$HD\left(X,Y\right)=\max\left\{\max_{x\in X}\;\min_{y\in Y}d\left(x,y\right),\max_{y\in Y}\;\min_{x\in X}d\left(x,y\right)\right\}$$

In addition to overlap and boundary metrics (DSC, Jaccard, Hausdorff), we quantify probability calibration and uncertainty utility using the Expected Calibration Error (ECE), Uncertainty Calibration Error (UCE), Brier score, and Negative Log-Likelihood (NLL).

Expected Calibration Error (ECE) quantifies how closely predicted confidences match observed accuracy. Uncertainty Calibration Error (UCE) measures how well a model’s predicted uncertainty matches its observed error.


$$ECE=\overset{M}{\underset{m=1}{\sum }}\frac{\left|B_{m}\right|}{n}\left|accuracy\left(B_{m}\right)-confidence\left(B_{m}\right)\right|$$

$$UCE=\overset{M}{\underset{m=1}{\sum }}\frac{\left|B_{m}\right|}{n}\left|error\left(B_{m}\right)-uncertainity\;score\left(B_{m}\right)\right|$$

Here, B is used to represent “bins” and m is the bin number, while n represents the total number of evaluated predictions.

Brier score measures the mean squared error of predicted probabilities against the true outcomes.


$$Brier\;Score=\frac{1}{n}\overset{n}{\underset{i=1}{\sum }}{\left(p_{i}-x_{i}\right)}^{2},range\left[0,1\right]$$

Here, n is the total number of evaluated predictions, $p_{i}$ is predicted probabilities, $y_{i}$ is the true outcome.

Negative Log-Likelihood (NLL) measures how much probability a model assigns to the true class, averaged over samples.


$$\mathrm{Negative}\;\log-\mathrm{Likelihood}\;\left(\mathrm{NLL}\right)=-\frac{1}{n}\overset{n}{\underset{i=1}{\sum }}\log P_{\theta }\left(y_{i}|x_{i}\right)$$

Here, n is the total number of evaluated predictions, $y_{i}$ is prediction, $x_{i}$ is the true outcomes.

### 2.5 Statistical analysis

The Wilcoxon signed-rank test was selected because it is a non-parametric test designed for paired data, which is suitable for comparing segmentation performance metrics (e.g., Dice coefficients) of different models evaluated on the same set of subjects. Unlike parametric alternatives (e.g., paired t-tests), the Wilcoxon test does not assume normality of the performance distributions, which is important since metrics such as Dice coefficients and Hausdorff distances are often non-normally distributed and bounded. To address the issue of conducting multiple comparisons across different tumor subregions, we applied the Bonferroni correction, which provides a conservative adjustment to control the family-wise error rate. This combination ensures a robust and statistically sound evaluation of performance differences between models in our study.

## 3 Results

### 3.1 Follow-up MRI segmentation performance comparison

The GBSUN model was benchmarked against previous studies that performed segmentation on glioblastoma (GBM) using preoperative, postoperative, or follow-up MRI scans. All results are reported using the same test set for all models, with the exception of those by Helland et al. (2023) and BraTumIA (Meier et al., 2016). Helland et al. did not develop a new model but assessed the performance of previously established nnU-Net and AGU-Net models using early postoperative MRIs. Additionally, we were not able to locate the source code or pre-trained model for the BraTumIA framework; however, their manuscript indicates that they evaluated their model on preoperative, postoperative, and follow-up MRIs. Consequently, we relied on the Dice Similarity Coefficients (DSC) reported in their respective studies for these models.

The GBSUN model achieved average DSC scores of 0.833, 0.901, and 0.931 for the FHR, ER, and NENR regions, respectively, in follow-up MRI segmentation. Our proposed model consistently outperformed other models, with average improvements of 14.25, 19, and 24.38% for FHR, ER, and NENR (Table 1). The Jaccard Index (JI) and Hausdorff Distance (HD) values for the GBSUN model were 0.73, 0.85, 0.96, and 1.81, 0.56, 0.13 for FHR, ER, and NENR, respectively. The proposed model outperformed other models by an average of 37, 35, and 4% for FHR, ER, and NENR in JI, and by 32, 28, and 82% in HD evaluation metrics.

**Table 1:** Segmentation performance comparison with other studies using Dice similarity score, Jaccard Index, and Hausdorff distance.

Model Name	MRIs were used in the original model training	Dice similarity score			Jaccard index			Hausdorff distance		
		FHR	ER	NENR	FHR	ER	NENR	FHR	ER	NENR
GBSUN	Follow-up	0.833	**0.901**	**0.931**	0.76	0.85	**0.96**	1.81	0.56	**0.13**
Helland et al. (2023)	Early postoperative	X	X	X	X	X	X	X	X	X
BraTumIA (Meier et al., 2016)	Pre/postoperative, follow-up	X	0.23	0.63	X	X	X	X	X	X
2D U-Net (Dong et al., 2017)	Preoperative	0.74	0.77	0.67	0.55	0.73	0.57	3.19	0.55	3.13
3D-Unet (Wang et al., 2019)	Preoperative	0.68	0.84	0.83	0.68	0.84	0.83	2.34	1.31	0.200
3D U-Net (self-ensembled & deeply supervised) (Henry et al., 2021)	Preoperative	0.80	0.74	0.73	0.80	0.74	0.73	1.94	0.56	0.67
3D Dilated Multi-Fiber Network (Chen et al., 2019)	Preoperative	0.79	0.89	0.82	0.79	**0.89**	0.82	2.32	**0.53**	0.55
Knowledge Distillation (Lachinov et al., 2020)	Preoperative	**0.84**	0.74	0.75	**0.84**	0.74	0.75	**1.5**	0.56	0.22
ResUNet (Zhang et al., 2017)	Preoperative	0.51	0.73	0.54	0.51	0.73	0.54	3.02	0.85	1.70
ResNet (Zhang et al., 2017)	Preoperative	0.71	0.55	0.57	0.71	0.55	0.57	4.56	9.32	6.98
FCNN (Zhang et al., 2017)	Preoperative	0.58	0.66	0.51	0.58	0.66	0.51	3.56	1.26	2.63
Autoencoder Regularization/NvNet (Myronenko, 2019)	Preoperative	0.52	0.73	0.71	0.52	0.73	0.71	3.24	0.56	1.72
Inter-slice Context Residual Learning/ConResNe t (Inter-Slice Context Residual Learning, 2021)	Preoperative	0.53	0.74	0.52	0.53	0.74	0.52	2.84	0.56	2.86
Cascaded Anisotropic CNN (Wang et al., 2018)	Preoperative	0.81	0.74	0.77	0.81	0.74	0.77	2.13	0.56	0.45
3D U-Net with Attention (Nodirov et al., 2022)	Preoperative	0.74	0.86	0.87	0.25	0.28	0.02	1.81	0.67	0.59
SegNet (Badrinarayanan et al., 2015)	Preoperative	0.30	0.34	0.05	0.25	0.28	0.02	4.01	4.47	8.28
nnU-ne t (Isensee et al., 2021)	Preoperative	0.48	0.56	0.44	0.42	0.51	0.39	3.25	1.66	3.30
Swin-Unet (Cao et al., 2023)	Preoperative	0.67	0.81	0.56	0.64	0.79	0.46	2.67	0.55	6.01
UNETR (Hatamizadeh et al., 2022)	Preoperative	0.59	0.78	0.69	0.58	0.76	0.68	3.33	0.55	6.50
nnU-net (Isensee et al., 2021)	Follow-up	0.80	0.76	0.29	0.77	0.71	0.22	2.24	0.55	7.56
Swin-Unet (Cao et al., 2023)	Follow-up	0.65	0.67	0.15	0.63	0.65	0.1	2.65	0.86	8.18

‘X’ means the model was not evaluated. Bold values: best performance.

**Figure 4:**
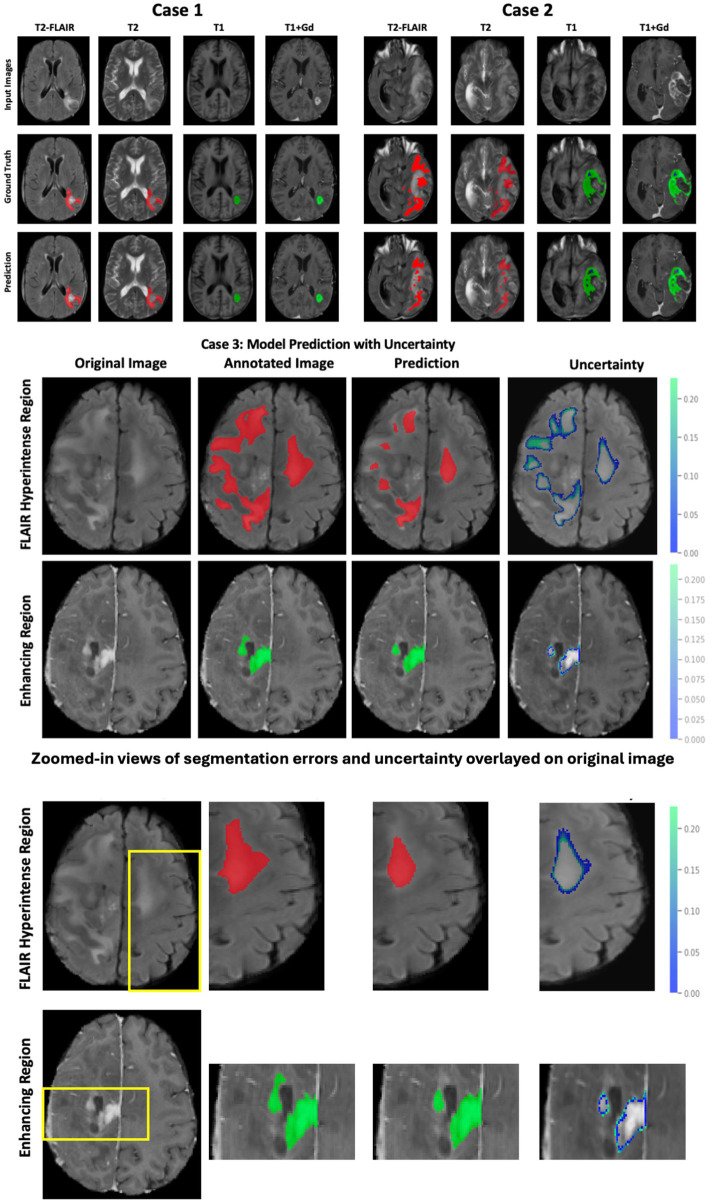
Brain tumor segmentation results using the proposed model. Top panel (Case 1 and Case 2): Input MRI modalities, corresponding ground truth annotations (red: FLAIR hyperintense region; green: enhancing tumor; blue: non-enhancing central necrosis), and model predictions. Middle panel (Case 3): Predictions with associated uncertainty maps for the FLAIR hyperintense region (top row) and enhancing region (bottom row), where uncertainty values highlight areas with a higher likelihood of misclassification. Bottom panel: Zoomed-in views of selected regions of interest (ROIs) demonstrate segmentation errors more clearly. In these magnified panels, overlays show mismatches between ground truth and prediction, as well as uncertainty contours highlighting boundaries prone to misclassification.

Figure 4 illustrates the follow-up model performance on two cases: Case 1, a well-performing case, and Case 2, a case with underperformance. The DSC for case 1 is 0.92 (FHR), 0.91 (ER), and 0.98 (NENR). In contrast, for Case 2, the DSC values are 0.76 (FHR), 0.82 (ER), and 0.85 (NENR). The shape and structure of the tumor subregions largely influence this performance variation. Case 1 exhibits a continuous shape for tumor subregions, whereas case 2 displays fragmented FHR and ER regions, which impact the model’s performance. Additionally, we report the uncertainty of the model prediction (Figure 3, case 3) to highlight the proposed model’s reliability and trustworthiness. The highest uncertainty was observed at the boundaries of the tumor subregions, indicating regions where segmentation errors are more likely to occur.

### 3.2 Ablation study

We conducted an ablation study to evaluate the contribution of each component: (i) the baseline 3D U-Net, (ii) 3D U-Net with transfer learning, (iii) 3D U-Net with Monte Carlo dropout, (iv) 3D U-Net with label smoothing, (v) 3D U-Net with test-time augmentation, (vi) 3D Bayesian U-Net, and (vii) the full GBSUN model. The results, presented in Table 2, show that transfer learning consistently improves segmentation performance across all tumor subregions. Meanwhile, the Bayesian component enhances calibration and robustness but yields lower Dice scores when applied in isolation (Table 2). Alternative uncertainty strategies, such as label smoothing and test-time augmentation, produced even lower DSCs, particularly for the enhancing and non-enhancing regions. In contrast, the full GBSUN model achieved the highest performance across all regions (0.833 ± 0.088, 0.901 ± 0.073, and 0.931 ± 0.065). The ablated version without bias correction, however, showed reduced performance, underscoring the importance of calibration. Taken together, these findings demonstrate that transfer learning and architectural refinements drive significant gains in segmentation accuracy, while the Bayesian component provides complementary benefits by enhancing reliability and interpretability when integrated into the full framework.

**Table 2:** GBSUN model’s improved DSC scores for tumor subregion segmentation.

Model Name	Fluid attenuation inversion recovery region (FHR) (Mean ± STD), 95% CI, *p*-value	Enhancing Tumor Region (ER) (Mean ± STD), 95% CI, *p*-value	Non-enhancing Region (NENR) (Mean ± STD), 95% CI, *p*-value
GBSUN	**(0.833** ± **0.088), [0.806, 0.851], 0.0132**	**(0.901** ± **0.073), [0.869, 0.910], 0.0004**	**(0.931** ± **0.065), [0.887, 0.928], 0.0004**
3D U-Net	(0.729 ± 0.101), [0.697, 0.750], 0.0004	(0.852 ± 0.078), [0.819, 0.862], 0.0008	(0.879 ± 0.074), [0.830, 0.877], 0.0004
3D U-Net with transfer learning	(0.803 ± 0.093), [0.774, 0.823], 0.9090	(0.898 ± 0.059), [0.872, 0.905], 0.0004	(0.865 ± 0.095), [0.801, 0.861], 0.0367
3D U-Net with Monte Carlo dropout	(0.757 ± 0.104), [0.725, 0.779], 0.0008	(0.844 ± 0.132), [0.786, 0.860], 0.2043	(0.832 ± 0.109), [0.760, 0.827], 0.740
3D U-Net with label smoothing	(0.760 ± 0.134), [0.718, 0.788], 0.0079	(0.715 ± 0.221), [0.619, 0.744], 0.0004	(0.574 ± 0.494), [0.247, 0.557], 0.0004
3D U-Net with test time augmentation	(0.766 ± 0.145), [0.720, 0.796], 0.0307	(0.702 ± 0.209), [0.613, 0.730], 0.0004	(0.171 ± 0.254), [0.000, 0.164], 0.0004
3D Bayesian U-Net	(0.752 ± 0.154), [0.703, 0.784], 0.0075	(0.860 ± 0.215), [0.664, 0.784], 0.0231	(0.532 ± 0.499), [0.204, 0.517], 0.0004
GBSUN without bias correction	(0.761 ± 0.338), [0.659, 0.834], 0.2247	(0.854 ± 0.309), [0.720,0.893],0.8994	(0.921 ± 0.192), [0.794,0.913], 0.0831
3D U-Net without bias correction	(0.679 ± 0.111), [0.655, 0.750], 0.0004	(0.789 ± 0.058), [0.819,0.862], 0.0023	(0.799 ± 0.084), [0.810,0.847], 0.0005

Bold values: best performance.

Additionally, Table 2 illustrates the comparative performance of the proposed GBSUN model against seven 3D U-Net baselines on follow-up MRIs across three glioblastoma sub-regions: FHR, ER, and NENR. For each method and region, we report the subject-level mean ± standard deviation (SD) of DSC, a 95% bias-corrected and accelerated (BCa) bootstrap confidence interval, and a bootstrap *p*-value for the null hypothesis that the mean DSC equals 0.80 (two-sided). GBSUN achieves the highest mean DSC in all three sub-regions: FHR 0.833, ER 0.901, NENR 0.931 with 95% BCa CIs entirely above the 0.80 benchmark [0.806–0.851], [0.869–0.910], [0.887–0.928] and corresponding *p*-values ≤0.013, indicating performance significantly exceeding 0.80 across the board. Relative to a plain 3D U-Net, the absolute gains are +0.104 (FHR), +0.049 (ER), and +0.052 (NENR). Even against the strongest non-GBSUN variants, GBSUN still leads. For instance, FHR + 0.030 over 3D U-Net + transfer learning (0.803), and a clear margin on the most challenging NENR class (0.931 vs. the next best baseline 0.879). GBSUN is also more consistent across subjects: its standard deviations are among the smallest, especially for NENR (±0.065), and its CIs are relatively tight (e.g., NENR width ≈ 0.041). In contrast, several alternatives either fail to meet the 0.80 threshold (e.g., label smoothing, test-time augmentation, 3D Bayesian U-Net on NENR) or are inconclusive with CIs that cross 0.80 (e.g., MC-dropout on ER/NENR; transfer learning on FHR). Finally, the “GBSUN without bias correction” ablation shows noticeable drops in FHR and ER means, as well as a loss of significance, underscoring the importance of the intensity bias-field correction in our preprocessing pipeline. Overall, GBSUN is the only method that consistently meets the clinical quality bar across all sub-regions, with strong and well-calibrated performance.

**Figure 5:**
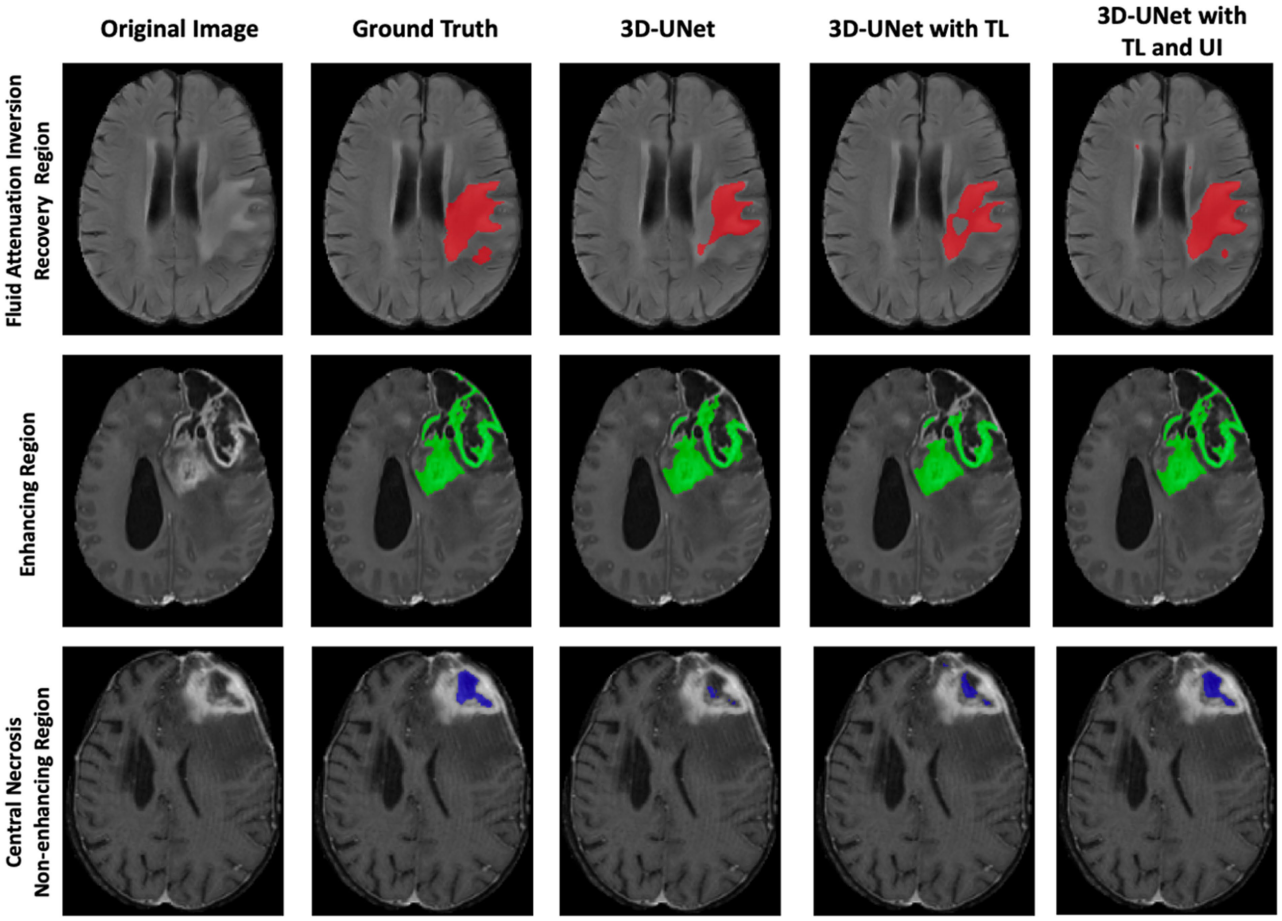
Comparison of 3D-UNet models for brain tumor segmentation. Columns show the original MRI, ground truth, baseline 3D-UNet, 3D-UNet with transfer learning (TL), and 3D-UNet with TL and uncertainty information (UI). Rows correspond to the FHR region (red), ER region (green), and NENR region (blue). Incorporating TL improves boundary delineation, while TL combined with UI reduces false positives and enhances agreement with ground truth. These results demonstrate that transfer learning and uncertainty information together yield more reliable and generalizable tumor segmentation across multiple patients’ MRIs.

Figure 5 visually demonstrates the improved performance using transfer learning and uncertainty information for three cases. In each case, some regions are misclassified by either the 3D U-Net or the 3D U-Net with transfer learning. However, the 3D U-Net with uncertainty and transfer learning consistently provides more accurate segmentation and reduces misclassification compared to the other models.

Moreover, to evaluate the contribution of bias field correction, we compared the full GBSUN pipeline with a variant where N4 bias field correction was omitted (Table 2). Without bias correction, Dice scores decreased across tumor subregions (e.g., FHR: 0.761 vs. 0.833; ER: 0.854 vs. 0.901; NENR: 0.921 vs. 0.931) and showed larger variability, particularly in the FHR and enhancing tumor regions. This demonstrates that N4 correction is especially beneficial in our multi-scanner dataset, as it mitigates scanner-related intensity inhomogeneities and harmonizes tissue contrast. The improvements confirm that bias correction not only stabilizes performance but also reduces variance, supporting its inclusion as a critical preprocessing step for robust segmentation.

Finally, we assessed robustness by measuring the change in Dice similarity coefficient (ΔDSC) under clinically plausible bias perturbations to emulate distribution shift. The Bayesian model exhibited smaller degradation than the non-Bayesian counterpart (ΔDSC: FHR = 0.05, ER = 0.07, NENR = 0.14), and the complete GBSUN pipeline showed the smallest ΔDSC overall (FHR = 0.07, ER = 0.05, NENR = 0.01), indicating superior robustness to acquisition and preprocessing variability. As summarized in Table 2, we compared the full GBSUN pipeline with a 3D U-Net baseline, each evaluated with and without N4 bias-field correction. Omitting bias correction reduced Dice scores across all tumor subregions (FHR, ER, and NENR).

### 3.3 Statistical analysis

We conducted the Wilcoxon signed-rank test to compare the Dice Similarity Coefficient (DSC) values of different models against GBSUN across all test samples for various regions reported in Table 3 (with 95% confidence interval). A *p*-value of less than 0.05 indicates that the differences in model performance are statistically significant.

**Table 3:** The Wilcoxon signed-rank test on the DSC values between the GBSUN model and other models.

Model Name	FHR (*p*-value)	ER (*p*-value)	NENR (*p*-value)
2D U-Net	7.72e-14	1.82e-12	3.30e-19
3D-Unet	3.84e-08	2.28e-10	1.38e-34
3D U-Net (self-ensembled & deeply supervised)	3.78e-05	8.07e-12	3.95e-31
3D Dilated Multi-Fiber Network	1.15e-05	4.12e-06	2.34e-23
Knowledge Distillation	7.25e-03	8.07e-12	9.39e-30
ResUNet	8.53e-25	2.40e-12	1.04e-44
ResNet	1.00e-09	1.60e-22	7.23e-41
FCNN	9.09e-19	2.49e-16	6.51e-50
Autoencoder Regularization/NvNet	6.36e-24	2.40e-12	2.23e-32
Inter-slice Context Residual Learning/ConResNet	4.77e-23	8.07e-12	4.02e-48
Cascaded Anisotropic CNN	1.28e-04	8.07–12	3.22e-28
3D-Unet with Attention	3.84e-08	4.54e-07	1.09e-15
nnU-net	7.34e-34	5.91e-39	9.22e-35
SegNet	2.42e-27	6.17e-22	5.85e-48

The *p*-value shows the significance of DSC variation.

In addition, we conducted paired Wilcoxon signed-rank tests comparing GBSUN with each comparator model within the FHR, ER, and NENR regions, with Bonferroni adjustment for multiple comparisons (Supplementary Table 6). Adjusted *p*-value on the order of 10^−14^ to 10^−9^ provides robust evidence that the GBSUN model consistently and significantly outperforms the other models.

### 3.4 Calibration and uncertainty-aware performance

We further evaluated the reliability of GBSUN using complementary calibration and probabilistic accuracy metrics. The voxel-wise Uncertainty Calibration Error (UCE) was 0.007, indicating excellent agreement between predicted uncertainty and empirical error. UCE could not be computed for the 3D U-Net and its transfer-learning variant because these models produce point estimates rather than probabilistic predictions. UCE requires per-sample uncertainty scores to compare predicted uncertainty with empirical error; in their absence (i.e., with hard labels only), any constant surrogate collapses UCE to a trivial quantity that merely reflects overall error.

The mean voxel-wise Expected Calibration Error (ECE) was 0.007484 overall, with class-wise ECEs of 0.000434 (FHR), 0.004315 (ER), and 0.004459 (NENR), demonstrating consistently low miscalibration across classes (Supplementary Table 7). Proper scoring rules further substantiated these findings: the average Brier score and Negative Log-Likelihood (NLL) were 0.002645 and 0.060173 for FHR, 0.002499 and 0.058981 for ER, and 0.002503 and 0.037054 for NENR, respectively. By contrast, both the 3D U-Net and its transfer-learning variant yield substantially higher ECE, Brier, and NLL values, and the 3D Bayesian U-Net likewise lags behind GBSUN by a considerable margin. Taken together, these low UCE/ECE values and favorable Brier/NLL scores indicate that GBSUN’s voxel-wise probabilities are both well-calibrated and informative, supporting robust, trustworthy inference across all evaluated classes. Additionally, Bayesian components reduce performance variability; for example, GBSUN exhibits a small NENR DSC SD (±0.065) (Table 2), indicating more consistent case-level behavior, which is clinically valuable.

Moreover, we also generated a reliability curve to assess probability calibration of the GBSUN model (Supplementary Figure 4). Across panels, the curves closely track the diagonal, indicating good agreement between predicted probabilities and observed values. Consistent with the visual impression, Expected Calibration Error (ECE) is low in all cases—Overall = 0.007484, FHR = 0.000434, ER = 0.004315, and NENR = 0.004459—supporting that GBSUN’s voxel-wise probabilities are well calibrated.

Additionally, we evaluated “uncertainty-as-error-detector” performance by scoring each voxel with predictive variance/entropy and classifying voxels as incorrect (positive) versus correct (negative). Threshold-swept receiver-operating characteristics (ROCs) demonstrate that GBSUN consistently ranks errors more effectively than the non-Bayesian 3D U-Net (Supplementary Figure 5) for each class, the GBSUN ROC curves dominate—achieving higher true-positive rates at comparable false-positive rates and larger areas under the ROC (AUROC).

Finally, we evaluated the GBSUN model using a risk–coverage (RC) curve to assess how effectively its uncertainty ranking enables selective prediction (Supplementary Figure 6). The voxel-level RC curve exhibited the expected monotonic behavior: risk was lowest for the most-confident voxels and increased as coverage expanded, then gradually plateaued near full coverage, indicating that errors are concentrated in the low-confidence tail.

## 4 Discussion

In this study, we developed GBSUN (GlioBlastoma Segmentation and Uncertainty estimatioN), designed for accurate segmentation of follow-up MRIs. The GBSUN model effectively identifies various areas, including the Fluid Attenuation Inversion Recovery (FLAIR) region (FHR), the Enhancing Tumor Region (ER), and the Non-Enhancing Central Necrosis Region (NENR).

Accurate follow-up MRI segmentation plays a crucial role in planning surgery and evaluating treatment in glioblastoma (GBM) patients. It allows clinicians to monitor tumor progression, adjust treatment plans, and provide reliable data for clinical trials (Olar and Aldape, 2014). Additionally, it facilitates optimal tumor removal during surgery while minimizing damage to healthy brain tissue (Liu et al., 2023; Yabo and Heiland, 2024). In radiotherapy, precise segmentation ensures targeted radiation delivery to the tumor while sparing surrounding healthy tissue. However, achieving accurate segmentation in follow-up MRIs remains challenging due to factors such as 1) changes in tumor appearance over time, 2) variability in tumor shape and size, 3) artifacts that complicate the distinction between tumor and healthy tissue, and 4) partial volume effects and edema present in follow-up images.

Our design began from three empirical constraints of follow-up GBM MRI segmentation: (i) distribution shift between pre- and post-treatment anatomy (surgical cavity, scar, radiation changes), (ii) class imbalance and scarcity, especially for NENR (47/311 cases), and (iii) volumetric inference cost (four MRI channels, 3D U-Net). We required a method that provides pixel-wise epistemic uncertainty to flag boundary errors, integrates cleanly with a 3D U-Net, and keeps the parameter footprint near 1 × to remain deployable.

We considered two widely used practical Bayesian approximations, Monte Carlo (MC) dropout, and deep ensembles, alongside a variational Bayesian network with Flipout convolutional layers. Deep ensembles generally offer strong calibration but would require training, storing, and serving K independent 3D models and performing K full 3D forward passes per case (multiplying both training time and memory by K). MC dropout keeps a single model, but its uncertainty quality is sensitive to where dropout is inserted in encoder/decoder skip pathways, and it can interact unfavorably with normalization layers in segmentation pipelines.

Given these constraints, we selected a Bayesian 3D U-Net with Flipout variational convolutions in the decoder, trained with a KL term plus generalized Dice–focal loss. This design preserves a single-model footprint, yields posterior samples by weight perturbation at test time (tunable cost via T stochastic passes), and integrates naturally with our transfer-learning pathway for NENR. In ablations (Table 2), this Bayesian model outperformed the same 3D U-Net with MC dropout, particularly for FHR and NENR, and enabled our uncertainty-guided relabeling rule (case-specific threshold using background mean/variance, *λ* = 0.1) that further reduced false negatives. We did not train deep ensembles due to the K × compute/storage overhead for 3D volumes.

Bayesian component using Flipout layers uses a distribution for each learnable parameter in the model and enables posterior sampling during inference. Multiple stochastic forward passes generate voxel-wise uncertainty maps in addition to segmentation outputs. These maps quantify the variance of pixel-level predictions and assign uncertainty scores to each voxel, which significantly reduced misclassification rates across all tumor subregions, particularly in the FHR and NENR regions (Table 2), where boundaries are visually ambiguous. Compared with the Monte Carlo–dropout variant in Table 2, GBSUN outperforms across all subregions than MC dropout, especially in enhancing/non-enhancing regions. GBSUN’s transfer learning and targeted refinements, combined with a FlipOut-based Bayesian component, deliver both higher accuracy and calibrated, spatially localized uncertainty.

Our findings demonstrate significant improvements compared to the previous study in terms of the DSC across all evaluated regions—FHR, ER, and NENR—illustrating the effectiveness of our approach in accurately delineating tumor subregions in follow-up MRIs. The GBSUN model achieved DSC scores of 0.833, 0.901, and 0.931, representing average enhancements of 14.25, 19, and 24.38%, respectively, over prior models. The GBSUN model achieved a UCE (Uncertainty Calibration Error) of 0.007, demonstrating that predicted uncertainties are well aligned with actual error rates. This low value indicates reliable calibration, enabling uncertainty maps to effectively highlight regions that may require additional clinical attention.

A critical advantage of the GBSUN model is its ability to integrate transfer learning and uncertainty information. The introduction of Bayesian learning for uncertainty information provides valuable insights into the model’s reliability, particularly in boundary regions where segmentation errors are likely to occur. As shown in Figure 3, areas of higher uncertainty align with these critical boundaries, underscoring the need to consider uncertainty in clinical applications to enhance diagnostic accuracy. Additionally, case-specific threshold values for uncertainty calculations help to minimize the false negatives. This advancement is significant, highlighting our framework’s capability to enhance the accuracy of medical imaging analysis, a crucial factor in providing patients with precise diagnostic assessments and optimal treatment strategies.

In our comparative analysis (Table 1), GBSUN consistently outperformed existing methods across all tumor subregions. It achieved the highest Dice scores for the enhancing region and the non-enhancing/necrotic region, while also yielding the lowest Hausdorff Distance in the NENR, reflecting superior overlap accuracy and boundary delineation compared to advanced models such as the 3D Dilated Multi-Fiber Network and cascaded CNNs. Furthermore, transformer-based architectures, such as Swin-UNet and UNETR, as well as the benchmark nnU-Net, underperformed relative to GBSUN, particularly in capturing fine boundary details. Statistical validation with the Wilcoxon signed-rank test (Table 3) confirmed that these improvements are highly significant, with low *p*-values. Therefore, these results demonstrate that GBSUN delivers state-of-the-art performance with statistically robust gains, particularly in boundary regions where precise delineation remains a major clinical challenge.

Finally, we used 5-fold cross-validation to minimize the risk of overfitting on the smaller post-operative dataset during transfer learning. We utilized out-of-fold testing in this study because it provides a more accurate and generalizable measure for evaluating the performance of predictive models. By leveraging cross-validation, out-of-fold testing offers a more robust estimate of model performance, helping to mitigate overfitting. This approach ensures that the model is tested on different subsets of data, improving the reliability of the evaluation and enhancing generalization to unseen data. Moreover, out-of-fold testing allows for the full utilization of all available data for both training and testing, thereby maximizing the use of valuable information.

The challenges posed by small and disconnected regions, such as NENR, in follow-up MRIs are noteworthy. The GBSUN model’s ability to mitigate misclassification in these scenarios, as indicated by reduced DSC variation, highlights the robustness of our approach. By combining transfer learning with uncertainty information, we have improved accuracy and enhanced the model’s resilience to the unique challenges presented by GBM imaging. From a clinical perspective, the availability of uncertainty maps enhances interpretability by flagging regions of high uncertainty, such as tumor boundaries and areas with atypical tissue appearance, guiding radiologists to review these regions more carefully. This integration of uncertainty into segmentation outputs increases reliability, supports transparency in clinical decision-making, and enhances trust in the AI models.

A limitation of our study is that only a single follow-up scan was available for each patient, which precluded assessment of longitudinal consistency and temporal reproducibility of our method. Future studies incorporating multiple follow-up scans will be essential to validate the stability and robustness of the proposed approach over time.

We carefully searched for publicly available post-treatment glioma MRI datasets suitable for external validation. To the best of our knowledge, no such datasets with complete multi-modal MRI (FLAIR, T1, T1 + Gd, T2) and annotated segmentations are currently available. We therefore acknowledge this as a limitation of our study. Once curated, publicly released post-treatment datasets become available, we plan to evaluate our trained model on them to strengthen external generalizability.

While our findings are promising, it is essential to consider model performance variability and the need for further validation. Future studies should focus on evaluating the GBSUN model across diverse datasets, including a broader range of MRI modalities and tumor stages, to establish its generalizability. Additionally, exploring the integration of other imaging modalities, such as PET or CT, could provide a more comprehensive view of tumor biology and improve segmentation outcomes.

## 5 Conclusion

We introduced an enhanced end-to-end deep learning model, GBSUN, designed for follow-up MRIs, which offers more accurate and automated segmentation of glioblastoma (GBM) tumors. The model excels in measuring tumor subregions and signals while providing pixel-level uncertainty estimates. GBSUN represents a significant advancement in tumor segmentation, offering a reliable tool for clinicians managing GBM. By leveraging transfer learning and incorporating uncertainty information, our approach not only improves segmentation accuracy but also boosts confidence in the clinical utility of these models. As we continue refining these methods, the potential for better patient outcomes through precise imaging and targeted therapies becomes increasingly achievable.

## Data Availability

The data analyzed in this study is subject to the following licenses/restrictions: the datasets used and/or analyzed during the current study are available from the corresponding author on reasonable request. Requests to access these datasets should be directed to shayan.shams@uth.tmc.edu.
