# Supplementary material for: A Bayesian deep segmentation framework for glioblastoma tumor segmentation using follow-up MRIs
Source: Front Neuroimaging. 2025 Oct 23;4:1630245. doi: 10.3389/fnimg.2025.1630245 (PMC12588840; doi:10.3389/fnimg.2025.1630245)
Supplement: Supplementary file 1 [file Data_sheet_1.docx]

# **Supplementary Tables, Figures, Text, and References**

**SUPPLEMENTARY TABLES**

**Supplementary Table 1:** The patients’ demographic information of institutional dataset

| **Number of cases: 311 follow-up MRIs** | |
| --- | --- |
| Gender | Male: 193, Female:118 |
| Age | 18-40 years: 26, 40-60 years: 96, Over 60 years: 189 |
| Race | Caucasian: 194, Hispanic: 33, African American: 22, Asian: 8, Native American: 2, Other: 52 |

**Supplementary Table 2:** Age distribution across the dataset

|  | **18-40 years** | **40-60 years** | **Over 60 years** |
| --- | --- | --- | --- |
| Overall Data | 10% | 30% | 60% |
| Training Data | 7% | 30% | 63% |
| Validation Data | 13% | 32% | 56% |
| Test Data | 12% | 34% | 53% |

**Supplementary Table 3A: The acquisition parameters for each MRI sequence using GE 3 Tesla Primier**

| PLANE | Voxels | TR` | TP | Flip | Ti | Slice TK | FOV | SENSE | Matrix | Nex | Time |
| --- | --- | --- | --- | --- | --- | --- | --- | --- | --- | --- | --- |
| T2-FLAIR | $0.6\times0.8$ | 10000 | 130 | na | 2569 | $4mm/0$ | $240\times192$ | $P2$ | $380\times280$ | 1 | 3.11min |
| T2 | $0.6\times0.8$ | 8214 | 120 | na | na | $4mm/0$ | $240\times192$ | $P2$ | $420\times320$ | 1 | 2.03min |
| T1 | $0.6\times0.8$ | 39037 | 24 | na | 972 | $4mm/0$ | $240\times192$ | $P2\times S 1.3cs$ | $420\times320$ | 1 | 2>10 min |
| T1+Gd | $0.9 \times0.9 iso IR900$ | 4.5 | 1.8 | 8 | na | $1.0mm$ | $240\times240$ | $P2\times S 1.3cs$ | $260\times260$ | 1 | 3.30min |

**Supplementary Table 3B: The acquisition parameters for each MRI sequence using Philips 3 T Ingenia**

| PLANE | Voxels | TR` | TP | Flip | Ti | Slice TK | FOV | SENSE | Matrix | Nex | Time |
| --- | --- | --- | --- | --- | --- | --- | --- | --- | --- | --- | --- |
| T2-FLAIR | $0.5x 0.6$ Fat Sat | 9000 | 100 | 90 | 2500 | $5mm/1mm$ | $240\times175$ | $P2$ | $400\times270$ | 1 | 1.5min |
| T2 | $0.75\times0.84$ | 3354 | 100 | na | na | $3mm/0$.03 | $240\times240$ | $S 1.7$ | $320\times238$ | 1.5 | 6.43min |
| T1 | $0.8\times0.98$ | 2000 | 20 | 90 | 2000 | $5mm/1mm$ | $240\times240$ | $p1.6$ | $300\times246$ | 1 | 3.54min |
| T1+Gd | $1.0 \times1.0 iso, IR 950$ | 7.9 | 3.5 | 8 | na | $1.0mm$ | $240\times240$ | $P1.5\times S 1.3$ | $240\times240$ | 1 | 5.37min |

TR: Repetition Time, TP: Echo Time, Ti: Inversion Time, Slice TK: Slice Thickness, FOV: Field of View, Nex: Number of Excitations

**Supplementary Table 4:** Segmentation performance comparison with other studies using Dice Similarity Score with standard deviation.

|  |  | Dice Similarity Score | | |
| --- | --- | --- | --- | --- |
| Model Name | MRIs used in original model training | FHR | ER | NENR |
| GBSUN | Follow-up | 0.833$\pm$0.088 | 0.901$\pm$0.073 | 0.931$\pm$0.065 |
| Helland et al.(Helland et al., 2023) | Early postoperative | X | X | X |
| BraTumIA(Meier et al., 2016) | Pre/postoperative, follow-up | X | 0.23 | 0.63 |
| 2D U-Net(Dong et al., 2017) | Preoperative | 0.74$\pm$0.14 | 0.77$\pm$0.20 | 0.67$\pm$0.25 |
| 3D-Unet(Wang et al., 2019) | Preoperative | 0.68$\pm$0.13 | 0.84$\pm$0.219 | 0.83$\pm$0.37 |
| 3D U-Net (self-ensembled & deeply supervised)(Henry et al., 2021) | Preoperative | 0.80$\pm$0.14 | 0.74$\pm$0.21 | 0.73$\pm$0.35 |
| 3D Dilated Multi-Fiber Network(Chen et al., 2019) | Preoperative | 0.79$\pm$0.11 | 0.89$\pm$0.22 | 0.82$\pm$0.35 |
| Knowledge Distillation(Lachinov et al., 2020) | Preoperative | 0.84$\pm$0.16 | 0.74$\pm$0.23 | 0.75$\pm$0.34 |
| ResUNet(Zhang et al., 2017) | Preoperative | 0.51$\pm$0.10 | 0.73$\pm$0.21 | 0.54$\pm$0.35 |
| ResNet(Zhang et al., 2017) | Preoperative | 0.71$\pm$0.10 | 0.55$\pm$0.21 | 0.57$\pm$0.36 |
| FCNN(Zhang et al., 2017) | Preoperative | 0.58$\pm$0.14 | 0.66$\pm$0.23 | 0.51$\pm$0.30 |
| Autoencoder Regularization/  NvNet(Myronenko, 2019) | Preoperative | 0.52$\pm$0.11 | 0.73$\pm$0.22 | 0.71$\pm$0.37 |
| Inter-slice Context Residual Learning/  ConResNet(Inter-Slice Context Residual Learning for 3D Medical Image Segmentation, n.d.) | Preoperative | 0.53$\pm$0.11 | 0.74$\pm$0.21 | 0.52$\pm$0.37 |
| Cascaded Anisotropic CNN(Wang et al., 2018) | Preoperative | 0.81$\pm$0.16 | 0.74$\pm$0.23 | 0.77$\pm$0.40 |
| 3D U-Net with Attention(Nodirov et al., 2022) | Preoperative | 0.74$\pm$0.13 | 0.86$\pm$0.19 | 0.87$\pm$0.39 |
| SegNet(Badrinarayanan et al., 2015) | Preoperative | 0.30$\pm$0.13 | 0.34$\pm$0.22 | 0.05$\pm$0.41 |
| nnU-net(Isensee et al., 2021) | Preoperative | 0.48$\pm$0.14 | 0.56$\pm$0.21 | 0.44$\pm$0.37 |
| Swin-Unet((Cao et al., 2023)) | Preoperative | 0.67$\pm$0.12 | 0.81$\pm$0.22 | 0.56$\pm$0.32 |
| UNETR((Hatamizadeh et al., 2022)) | Preoperative | 0.59$\pm$0.13 | 0.78$\pm$0.24 | 0.69$\pm$0.42 |
|  |  |  |  |  |

**Supplementary Table 5:** Segmentation performance comparison with different lambda values using Dice Similarity Score.

| Value | FHR | ER | NENR |
| --- | --- | --- | --- |
| 0.05 | 0.594 | 0.785 | 0.961 |
| 0.07 | 0.760 | 0.853 | 0.961 |
| 0.09 | 0.796 | 0.872 | 0.961 |
| **0.1** | **0.833** | **0.901** | **0.931** |
| 0.2 | 0.813 | 0.887 | 0.961 |
| 0.4 | 0.766 | 0.852 | 0.961 |

**Supplementary Table 6:** The Wilcoxon signed-rank test on the DSC values between the GBSUN and other models. The p-value shows the significance of DSC variation.

| **Model Name** | **Fluid attenuation inversion recovery region (FHR)** | **Enhancing Tumor Region (ER)** | **Non-enhancing Region (NENR)** |
| --- | --- | --- | --- |
| 3D U-Net | 1.42e-14 | 1.42e-14 | 1.42e-14 |
| 3D U-Net with transfer learning | 1.42e-14 | 0.838 | 1.42e-14 |
| 3D U-Net with Monte Carlo dropout | 1.42e-14 | 2.78e-09 | 1.42e-14 |
| 3D U-Net with label smoothing | 2.84e-14 | 1.52e-11 | 1.15e-08 |
| 3D U-Net with test time augmentation | 3.38e-11 | 6.11e-13 | 1.42e-14 |
| 3D Bayesian U-Net | 9.09e-12 | 1.29e-09 | 1.80e-09 |
| GBSUN without bias correction | 0.02 | 0.02 | 0.01 |

**Supplementary Table 7:** The Uncertainty Calibration Error (UCE), Expected Calibration Error (ECE), Brier Score and Negative Log-Likelihood (NLL) of the proposed GBSUN and other models. (X- not applicable)

| Model Name | UCE (Overall) | ECE (Overall, FHR, ER, NENR) | Brier Score (FHR, ER, NENR) | NLL (FHR, ER, NENR) |
| --- | --- | --- | --- | --- |
| **GBSUN** | **0.007** | **0.007484, 0.000434, 0.004315, 0.004459** | **0.002645, 0.002499, 0.002503** | **0.060173, 0.058981, 0.037054** |
| 3D U-Net | X | 0.084999, 0.013194, 0.011553, 0.099072 | 0.148603, 0.096584, 0.023899 | 0.801802, 0.905233, 2.995732 |
| 3D U-Net with transfer learning | X | 0.032693, 0.089353, 0.014397, 0.014999 | 0.028312, 0.015390, 0.035876 | 1.578901, 1.603620,  3.283414 |
| 3D Bayesian U-Net | 0.038 | 0.073289, 0.025747, 0.026148, 0.021895 | 0.068869, 0.070861  0.048231 | 1.357078, 0.966662, 1.155295 |

**SUPPLEMENTARY FIGURES**


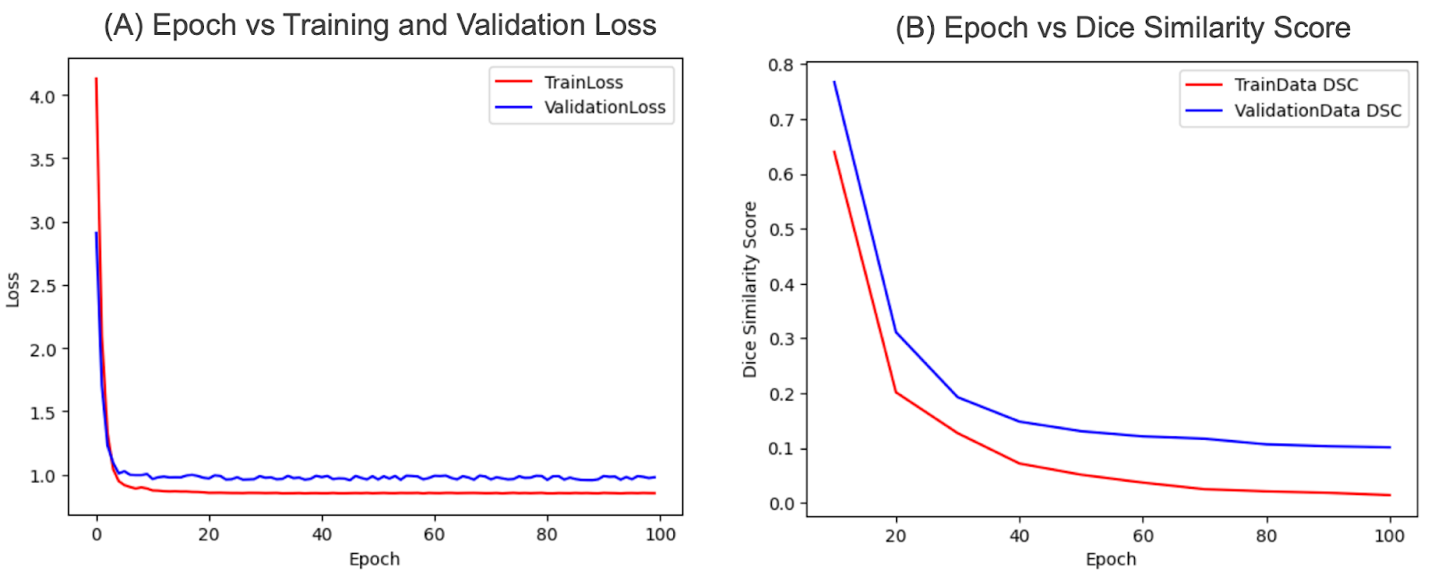


**Figure 1:** (A) Epoch vs training and validation loss curve, and (B) Epoch vs dice similarity score curve


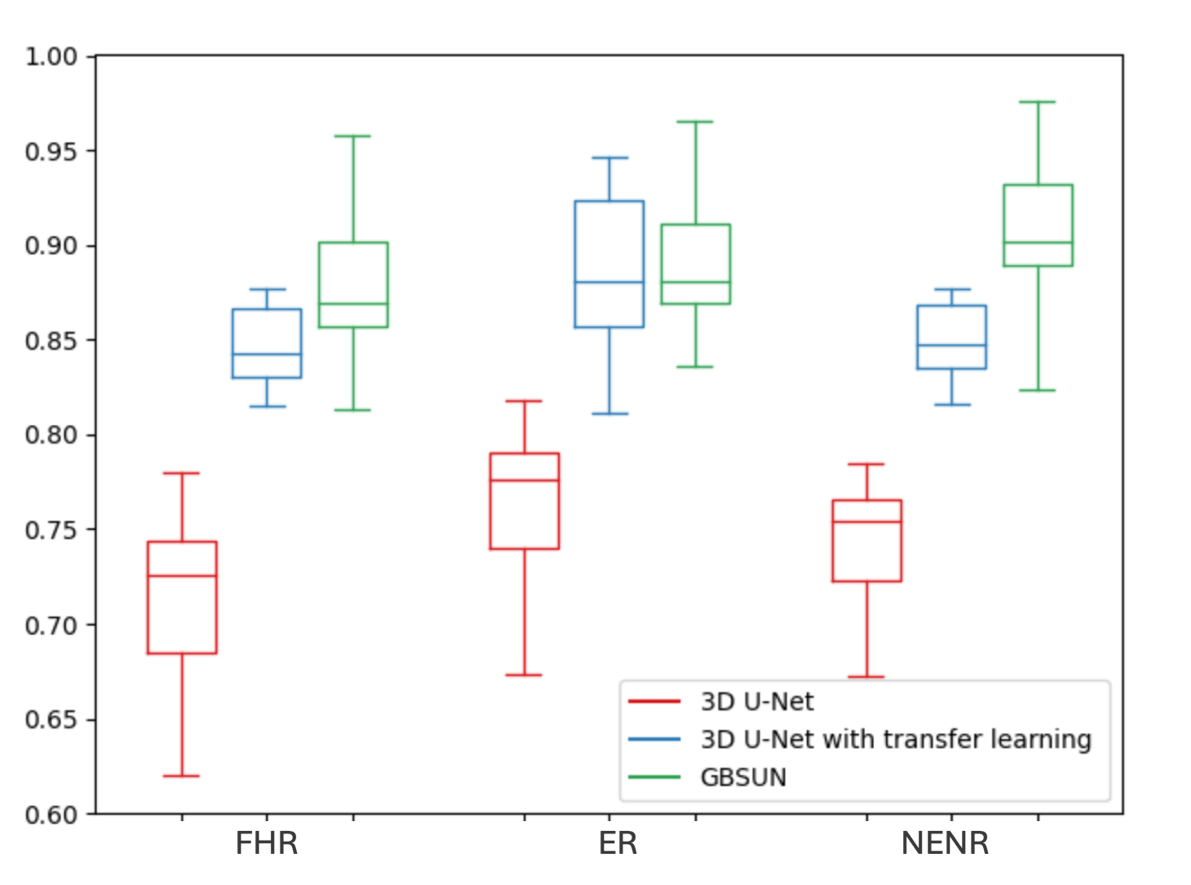


**Figure 2:** The proposed GBSUN model performance with original 3D U-Net and 3D U-Net with transfer learning.

**Model Interpretability**

To provide the model's interpretability, we selected one case to demonstrate the various visual features or patterns detected by each layer (Figure 3). Rather than showing only the strongest activation for each layer, we presented the top three activations. Our findings reveal that each filter detects distinct features or patterns, such as edges, textures, or more complex elements like shapes or object parts.


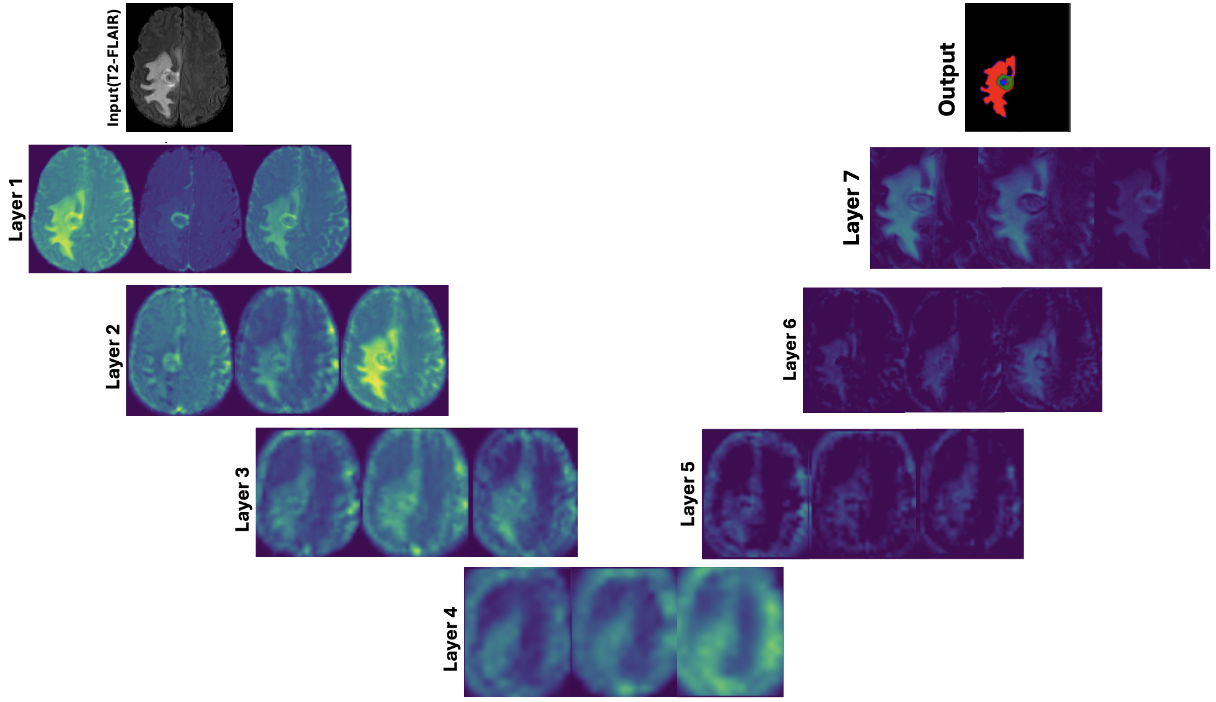


**Figure 3:** Feature maps for every convolution and deconvolution layers. Top 3 activations of each layer are displayed. The model identifies various tumor subregions as the final output: the fluid attenuation inversion recovery region (red), enhancing tumor region (green), and non-enhancing central Necrosis Regions (blue).


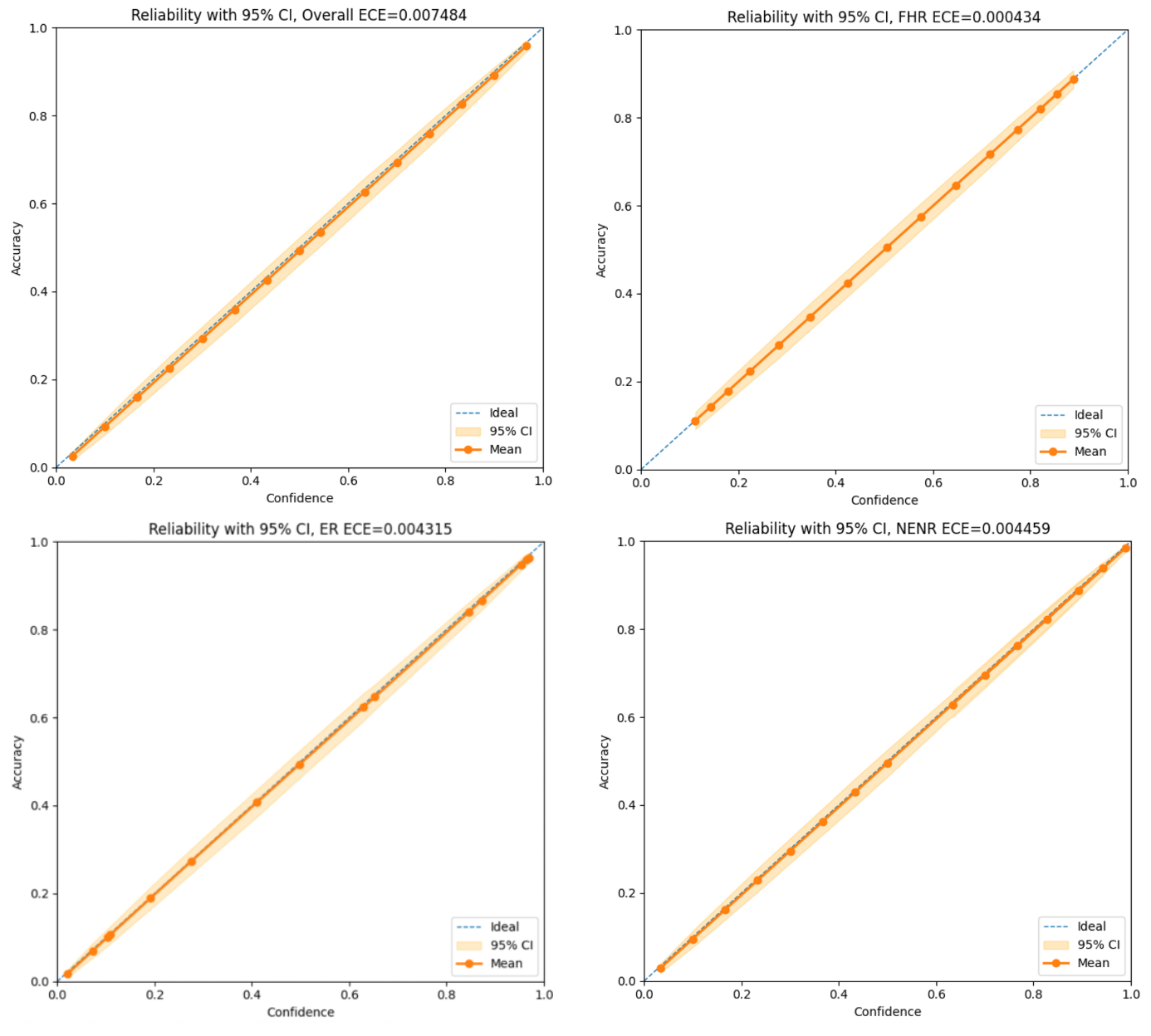
**Figure 4:** Reliability diagrams with 95% confidence intervals (Overall and by region: FHR, ER, NENR). The dashed line denotes perfect calibration (y = x); solid lines indicate the model’s mean calibration curve, with shaded bands showing 95% confidence intervals computed per confidence bin.


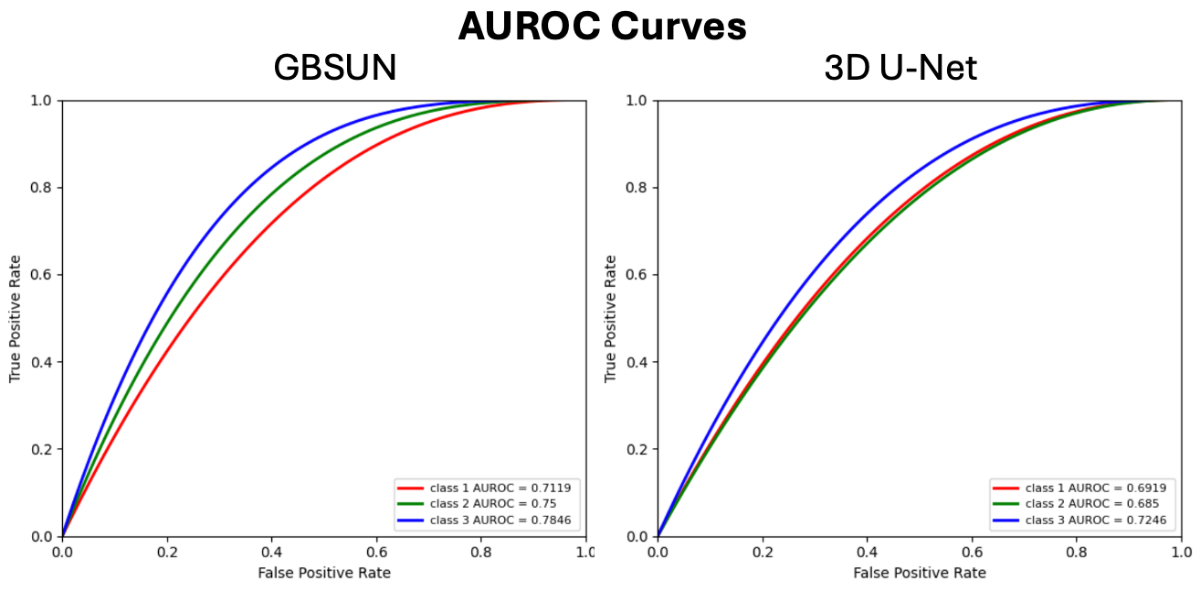


**Figure 5:** ROC curves for the three segmentation classes for comparing the performance of GBSUN and 3D U-Net models


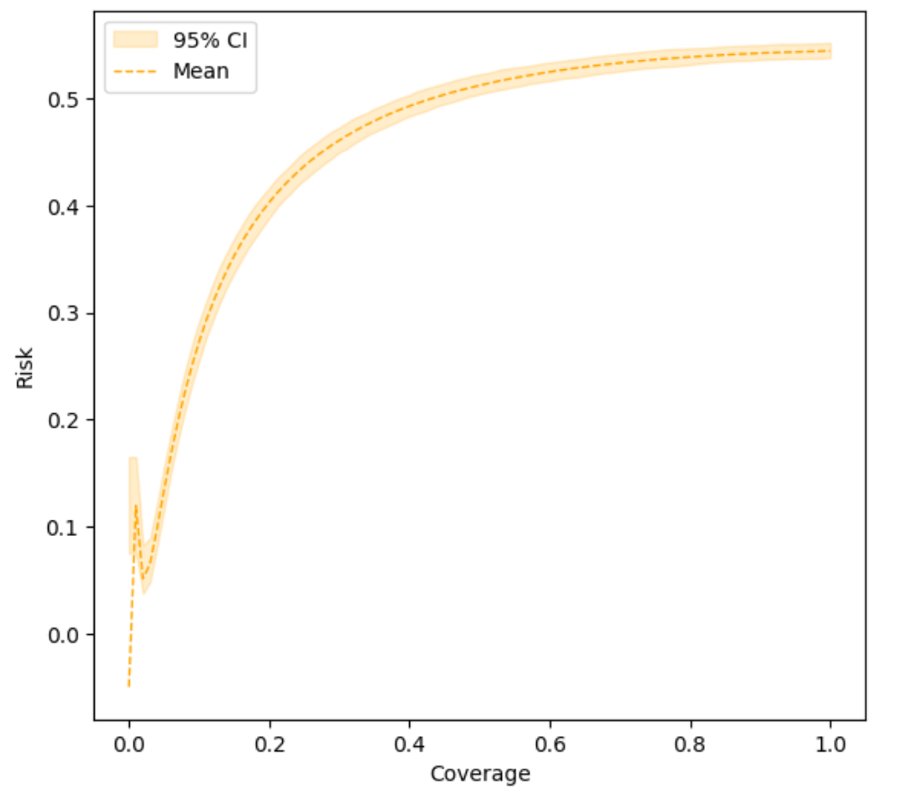


**Figure 6:** Risk-coverage curve for the proposed GBSUN model
